# Kinetic and Thermodynamic Interplay of Polymer-Mediated Liquid–Liquid Phase Separation for Poorly Water-Soluble Drugs

**DOI:** 10.1021/acs.molpharmaceut.4c00033

**Published:** 2024-05-20

**Authors:** Kaijie Qian, Lorenzo Stella, Fanjun Liu, David S. Jones, Gavin P. Andrews, Yiwei Tian

**Affiliations:** † School of Pharmacy, McClay Research Centre, Queen’s University Belfast, 97 Lisburn Road, Northern Ireland BT9 7BL, U.K.; ‡ School of Mathematics and Physics, Queen’s University Belfast, University Road, Belfast BT7 1NN, U.K.; § School of Chemistry and Chemical Engineering, Queen’s University Belfast, Stranmillis Road, Belfast BT9 5AG, U.K.

**Keywords:** liquid−liquid phase separation, supersaturation, amorphous solid dispersion, UV/vis spectrometer, hydrophobic interactions, ternary phase diagram

## Abstract

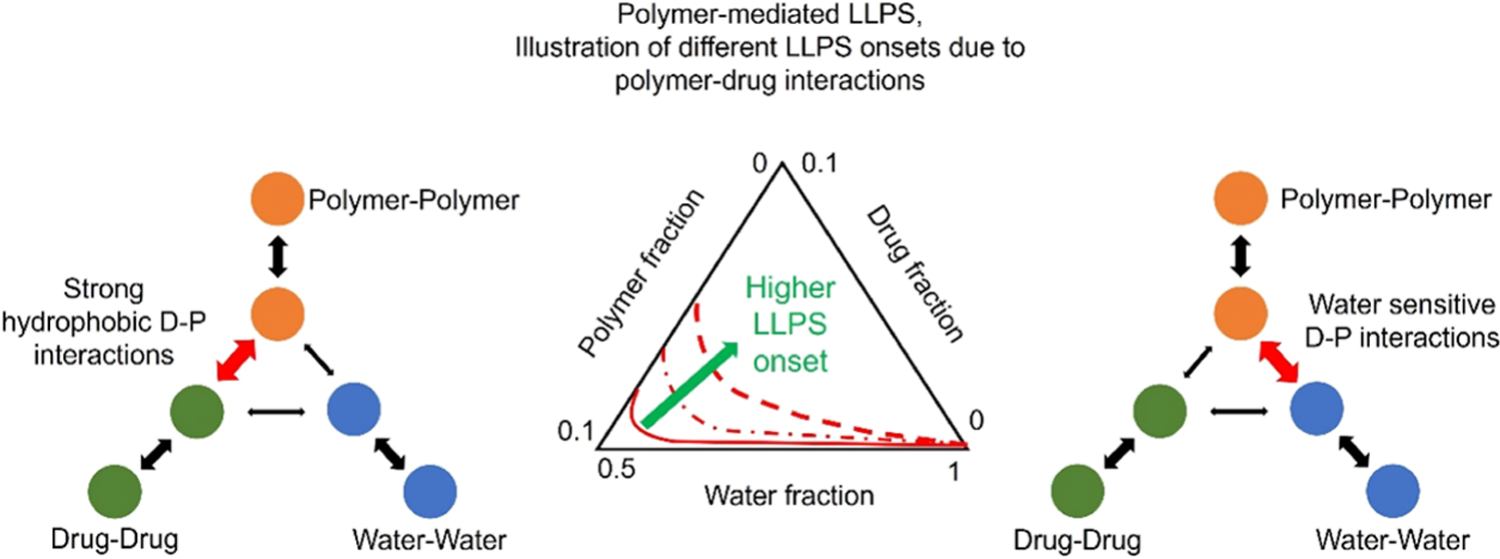

Understanding the interplay between kinetics and thermodynamics of polymer-mediated liquid–liquid phase separation is crucial for designing and implementing an amorphous solid dispersion formulation strategy for poorly water-soluble drugs. This work investigates the phase behaviors of a poorly water-soluble model drug, celecoxib (CXB), in a supersaturated aqueous solution with and without polymeric additives (PVP, PVPVA, HPMCAS, and HPMCP). Drug–polymer–water ternary phase diagrams were also constructed to estimate the thermodynamic behaviors of the mixtures at room temperature. The liquid–liquid phase separation onset point for CXB was detected using an inline UV/vis spectrometer equipped with a fiber optic probe. Varying CXB concentrations were achieved using an accurate syringe pump throughout this study. The appearance of the transient nanodroplets was verified by cryo-EM and total internal reflection fluoresence microscopic techniques. The impacts of various factors, such as polymer composition, drug stock solution pumping rates, and the types of drug–polymer interactions, are tested against the onset points of the CXB liquid–liquid phase separation (LLPS). It was found that the types of drug–polymer interactions, i.e., hydrogen bonding and hydrophobic interactions, are vital to the position and shapes of LLPS in the supersaturation drug solution. A relation between the behaviors of LLPS and its location in the CXB–polymer–water ternary phase diagram was drawn from the findings.

## 1 Introduction

Amorphous solid dispersion (ASD) has been well recognized as an enabling formulation strategy for the bioavailability enhancement of poorly water-soluble drugs.^1^ The high free energy state and disordered nature of these ASDs can lead to remarkably high water solubility and enhanced bioavailability. However, mechanistically understanding the ASD’s phase separation process during dissolution and storage is still challenging. In the dissolution of most amorphous drug formulations, various levels of supersaturation are anticipated, where the amount of dissolved drug is above the crystalline drug solubility in the respective aqueous media.^2^ The relationships between the maximum drug solubility in its crystalline form and the dynamics of the noncrystalline drug–water miscibility are critical to the phase behaviors of the amorphous formulation during dissolution. When the supersaturation of the drug solution is moderate, the system undertakes a classic nucleation pathway to reduce the free energy and form solid crystalline precipitates. Remarkably, if the level of supersaturation in the drug solution exceeds the spinodal boundary, drug-rich liquid or solid transient phases are often observed in the solution before the crystalline precipitates.^3^ The appearance of these transient phases suggests the mechanism of a different nucleation pathway for these amorphous formulations during dissolution, perhaps through liquid–liquid phase separation (LLPS).^4^ LLPS is a common phenomenon in which a fluid separates into solute-rich and solute-lean phases. LLPS occurs in cells,^5^ plays a vital role in infections,^6^ and is critical to the self-assembling of amphiphilic molecules.^7^ Thanks to high-resolution analytical techniques, the importance of LLPS has been widely investigated. Its impacts in the scientific fields of chemistry, biology, and pharmaceutics for the nucleation and crystallization of polymers, proteins, minerals, and small organic molecules are also discussed.^8−10^ Indeed, numerous studies have impressively illustrated the applications of LLPS in understanding and designing amorphous solids.^11,12^

In a typical binary phase diagram, binodal and spinodal curves govern the mixture’s phase behaviors. When the temperature and composition of the mixture sit within the binodal regions, the thermodynamic favored LLPS occurs. Interestingly, a recent study demonstrates that the liquid phases are likely the dynamic aggregates of clusters of the solute.^13^ Thus, at a given temperature, changing the composition of the mixture can result in the occurrence of LLPS in the solution, perhaps simply shifting the location of the mixture from outside the binodal boundary into the binodal region.^14^ Therefore, discussing LLPS is often accompanied by the nonclassical nucleation theory, suggesting the complex dynamic behaviors of these supersaturation drug solutions.^15−17^ The free energy barrier to form these metastable transient phases is smaller than that to form solid crystal nuclei. The condensed liquid or cluster is primarily formed from the original phase during LLPS and is suggested to be the precursor for further nucleation. Although many efforts have been made to elucidate the nonclassical nucleation pathway, this process has not been fully understood. The two-step mechanism is one possible hypothesis to describe the nonclassical nucleation pathway.^18^ The concentration and structure fluctuations are the two parameters accompanying the nuclei-forming process.^19^ With the formation of LLPS, the concentration and structural fluctuation can further decrease the energy barrier to form stable nuclei within the clusters of droplets.^20^ Additives and impurities are revealed to significantly affect the appearance and dynamics of the LLPS and the resulting different polymorphic forms.^21−23^ Additives can influence the nucleation of the solute from the solution by changing the Gibbs free energy landscape of the drug–polymer–water system, the position and width of the metastable zoom (miscibility gap), crystallization introduction time, and structure evolution of the clusters. In the case of ASD dissolution, polymers can be treated as the most essential additive in the supersaturated drug solution. The formation of nanosized liquid or solid transient phases is frequently observed in polymer-mediated drug–water–polymer ternary systems, suggesting its critical impact in maintaining the supersaturation of the drug solutions.^24,25^

Two main scenarios have been presented from the currently known cases of polymer-mediated LLPS in supersaturated solutions. In most cases, polymeric excipients have been reported not to change or to have a limited influence on the LLPS onset point (binodal curve). However, the concentration of polymers is in orders of magnitude higher than the drug in the aqueous media.^26,27^ Some other examples suggested that polymers can alter the LLPS onset point of the drug-water system.^28−30^ It is worth noting that the polymer types and concentrations were often randomly selected or fixed in these studies without a clear understanding of the boundaries of the drug–polymer–water ternary system. Thus, any systematic method to guide a polymer-mediated LLPS may help us improve our knowledge of this screening approach for polymer selections. In this work, the drug–polymer–water ternary phase diagrams were constructed for celecoxib (CXB)–water solutions with polymers of poly(vinylpyrrolidone) (PVP), poly(vinylpyrrolidone/vinyl acetate) (PVPVA), hydroxypropyl methylcellulose acetate succinate-M grade (HPMCAS-MF), and hydroxypropyl methylcellulose phthalate (HPMCP). The kinetics of LLPS were detected using the UV/vis spectroscopic method. The interplay between the kinetics of the mixing and thermodynamics of the ternary systems was discussed. The impacts of several critical parameters, such as the drug–polymer–water interaction parameters and the types of drug–polymer interactions, were discussed in relation to the positions of the resulting LLPS.

## 2 Materials and Methods

### 2.1 Materials

Celecoxib (CXB) was purchased from Kemprotec (Carnforth, U.K.). Poly(vinylpyrrolidone) (PVP) and poly(vinylpyrrolidone/vinyl acetate) E-635 (PVPVA) were obtained from Ashland (Kidderminster, U.K.). Hydroxypropyl methylcellulose acetate succinate-M grade (HPMCAS-MF) and hydroxypropyl methylcellulose phthalate (HPMCP) were donated by Shin-Etsu Chemical Company Ltd. (Tokyo, Japan). Methanol (MeOH) and phosphate-buffered saline (PBS) solution (including chemicals of sodium chloride, potassium chloride, sodium phosphate dibasic, and potassium phosphate monobasic) were purchased from Sigma-Aldrich Company Ltd. (Gillingham, U.K.). The purified water was obtained using a PKPD Millipore water purification system 7. Water resistivity was 18.2 MΩ·cm (Merck, U.K.). NMR deuterated solvent dimethyl sulfoxide-d_6_ (DMSO-*d*_6_) was purchased from Sigma-Aldrich Company Ltd. (Gillingham, U.K.).

### 2.2 Methods

#### 2.2.1 Solution ^1^H Nuclear Magnetic Resonance (NMR) Spectroscopy

The solution ^1^H NMR spectra investigated interactions between the CXB and polymers. One dimension ^1^H NMR spectra were collected using a Bruker Magnet System Ascend 400 MHz spectrometer (Bruker GmbH, Mannheim, Germany) with an acquisition time of 4 seconds, 2-second relaxation delay, 64 scans per sample at 25 °C. Pure CXB, PVP, PVPVA, HPMCAS-MF, HPMCP, and drug–polymer mixtures were dissolved in DMSO-*d*_6_. The drug concentration was fixed at 3 mg/mL, and the weight ratio of the drug and polymer was 1:5.

#### 2.2.2 Construction of Drug–Polymer–Water Ternary Phase Diagram

Drug–polymer–water ternary phase diagrams were constructed using a previously published method.^31^ Two approaches were utilized to obtain the binary Flory–Huggins interaction parameters. For water–polymer–drug systems, sorption isotherm experiments of water with the ingredients were collected using a DVS advantage system (Surface Measurement Systems, London, U.K.) at a temperature of 25 °C. Approximately 50–100 mg of ingredients were placed in a sample holder (mesh) within the DVS chamber. The sample environment humidity was then gradually increased from 0 to 90% RH at 10% RH intervals, using 120 min per step. The amount of water (in weight) absorbed into the sample at each water partial pressure was used to calculate the water–ingredient interaction parameter. Strong localized water–polymer bonding may occur for some partially frozen water–polymer systems such as HPMCAS. Hence, in this study, only a completely dried sample was used. Based on the DVS approach, the F–H interaction parameter may be derived using the activity of water in the mixture:
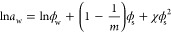
1where the ϕ is the volume fraction of water (ϕ_w_) or solute (ϕ_s_), and *m* is the molar volume ratio of the solute over water. The solvent in this study is the water; therefore, the change of water vapor partial pressure in the DVS tests can be used to define water activity (*a*_w_) in eq 1. For drug–polymer F–H interaction parameters at 25 °C, the Hildebrand solubility parameter approach was utilized for the calculations (Supporting Information).

#### 2.2.3 Ultraviolet/Visible (UV) Extinction Study

PVP, PVPVA, HPMCAS, and HPMCP polymers with a 1 mg/mL concentration were dissolved in the pH 7.4 PBS buffer at 37 °C. UV spectra of polymer solutions were scanned using the GENESYS 180 UV/Vis spectrophotometer (Thermo Fisher Scientific, Madison, USA) connecting with a fiber optic probe coupler in the range from 200 to 800 nm, with a scanning speed of 1 nm/s. In a typical procedure, 50–250 μL of CXB-MeOH stock solution (4 mg/mL) was gradually added into a 20 mL PBS solution (pH 7.4) using a syringe pump at various flow rates. The final CXB concentrations were 10, 20, 30, 40, and 50 μg/mL, in which 10 mg of polymer was predissolved.

#### 2.2.4 Determination of the CXB–Polymer–Water Ternary System LLPS Onset Concentration

The LLPS onset concentration point was determined using the ultraviolet (UV) extinction method. Polymers of PVP, PVPVA, HPMCAS-MF, and HPMCP with concentrations of 100, 500, or 1000 μg/mL were predissolved in PBS or codissolved with the CXB in the MeOH stock solution. Four mg/mL of the CXB stock solution in the 10 mL syringe was gradually added into 20 mL PBS solution using the Aladdin SyringeONE programmable syringe pump (AL-1000, Hitchin, U.K.) with various mixing rates of the stock solution and PBS. The mixing rates were controlled by altering the pumping rates at 3, 1, or 0.5 mL/h, generating the 50 μg/mL CXB solution in 30, 15, and 5 min, respectively. The PBS solution was stirred by a magnetic stirrer at 200 rpm, and a water bath was controlled at a constant temperature of 37 °C. Values of UV extinction were measured at the interval of 0.167 μg/mL CXB concentration until a clear extinction slope difference can be observed from the initial drug concentration, which indicates the formation of the drug-rich phase in the CXB–polymer–PBS ternary system. The UV extinction was determined at the wavelength confirmed (Supporting Information Figure S1), where UV absorption of CXB and polymer molecules can be insignificant.

#### 2.2.5 Verification of the LLPS of CXB–Polymer–Water via Cryo-TEM and Total Internal Reflection Fluorescence Microscopy

Cryogenic transmission electron microscopy (Cryo-EM, FEI, Thermo Fisher Scientific, Eindhoven, The Netherlands) was used to characterize the appearance of intransit nanoparticles/nanodroplets after the LLPS onset points. 3 μL of the liquid was pipetted onto a previously glow discharged, lacey carbon film EM grid, blotted for 1.2 s, and plunged frozen into liquid ethane using a Leica GP plunge freezer (Leica Microsystems, Wetzlar, Germany). The sample was kept at liquid nitrogen temperature while transferred to a Gatan 626 Cryotransfer holder (Gatan, Pleasanton, CA) and imaged using Cryo-EM. Images were acquired on a CETA camera (FEI, Thermo Fisher Scientific, Eindhoven, The Netherlands) using low-dose acquisition software. A total internal reflection fluorescence microscope (TIRFM, Lecia, Wetzlar, Germany) coupled with a 40X/0.85 NA HC PL APO objective lens was used to assess the appearance of the CXB–polymer LLPS phase. Pyrene was used as the hydrophobic fluorescence probe and dissolved with the CXB in methanol solution before pumping into the PBS using the 3 mL/h rate described above. For TIRFM fast acquisition, videos of the CXB–polymer suspensions were recorded using an Andor Zyla sCMOS camera with 4.2 megapixels (Oxford Instruments, Oxford, U.K.) at 100 ms per frame for 20 s. The excitation at 336 ± 40 nm and emission at 384 ± 40 nm were used. Samples were also recorded using the TIRFM with a polarizer at a crossed position for comparison. All videos were analyzed using ImageJ software (version 1.54f, National Institutes of Health, USA).

#### 2.2.6 Statistical Analysis

Data were analyzed using GraphPad Prism (version 9.0.0) and presented as mean ± standard deviation of three replicates. The statistical analysis was carried out using ordinary one- and two-way ANOVA. A significant difference was considered when *p* < 0.05.

## 3 Results and Discussions

### 3.1 Solution ^1^H NMR Spectroscopy

**Figure 1:**
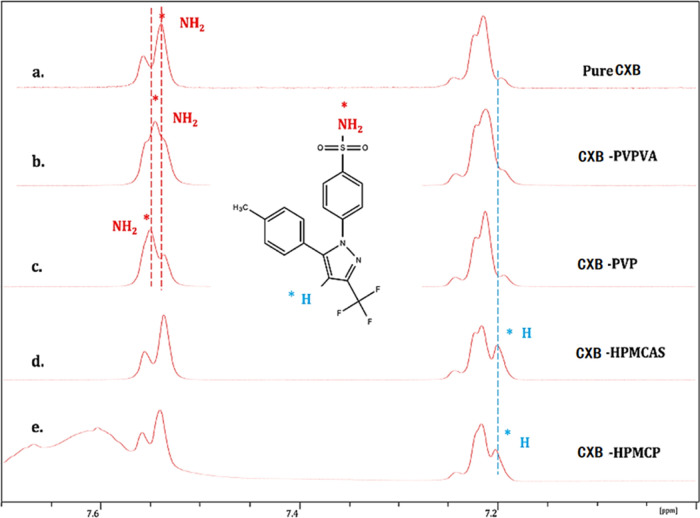
^1^H NMR spectra of (a) pure CXB and binary blends of (b) CXB–PVP, (c) CXB–PVPVA, (d) CXB–HPMCAS, and (e) CXB–HPMCP. The weight ratio of drug and polymers was 1:2. Red stars represent hydrogens on the –NH_2_, and blue stars represent hydrogen atoms on a pyrazole ring.

All solution NMR experiments were conducted in a nonaqueous environment to emphasize the drug–polymer interactions that may be more relevant to the ASD solids before rehydration (Figure 1). A nonaqueous solution in the NMR experiment can help differentiate the drug–polymer interactions for the drug–polymer–water ternary system before rehydration. It is important to investigate the relationship between the CXB–polymer interaction at a nonaqueous environment first and the subsequent dynamics of the LLPS. As suggested in this work, water can easily disrupt water senstive intermolecular interactions, such as the H-bonding between the drug and polymer. In contrast, hydrophobic interactions between the drug and the polymer are less affected during the dissolution of ASDs.^29,30^ We argue that if the drug–polymer interactions are mainly hydrophobic, then the influence of water on the LLPS onset point may be different from systems with other types of drug–polymer interactions.

The proton NMR results suggested that hydrogen bonding was formed within CXB–PVP and CXB–PVPVA combinations, supported by the downshift of hydrogens on the –NH_2_ (Figure 1a–c, red labels). The hydrogen atoms on the –NH_2_ group shifted to higher parts per million (ppm) in the presence of PVP, suggesting stronger hydrogen bonding between CXB and PVP than between CXB and PVPVA. This result was also reported previously in the literature. For example, IR spectroscopy indicated that the –VA groups of the PVPVA will not interact with CXB when forming ASDs.^32^ The critical interaction differences between CXB–PVP and CXB–PVPVA may also be highlighted by the level of determined drug–polymer glass transition temperatures deviating from the Gordon–Taylor equation predictions. Rask et al. found that the ASD of CXB–PVP has a more extended deviation of the glass transition temperatures rather than the CXB–PVPVA system, suggesting the CXB–PVP system generated a stronger interaction than the latter one.^33^

In comparison, hydrophobic interactions are the main form of intermolecular interactions for CXB–HPMCAS and CXB–HPMCP systems, as evidenced by the appearance of the lower ppm position of the hydrogen atoms in the pyrazole ring of the CXB (Figure 1d,e, blue labels). Similar upfield shifts have also been reported in the cases of CXB/hydroxypropyl-β-cyclodextrin and CXB/2,6-di-O-methyl-β-cyclodextrin systems.^34^ The upfield shift is caused by the shielding effect of oxygen atoms on the excipients, suggesting the CXB pyrazole ring was a critical hydrophobic group to interact with HPMCAS and HPMCP polymers in a nonaqueous environment.^35^

### 3.2 CXB–Polymer–Water Ternary Phase Diagrams

**Figure 2:**
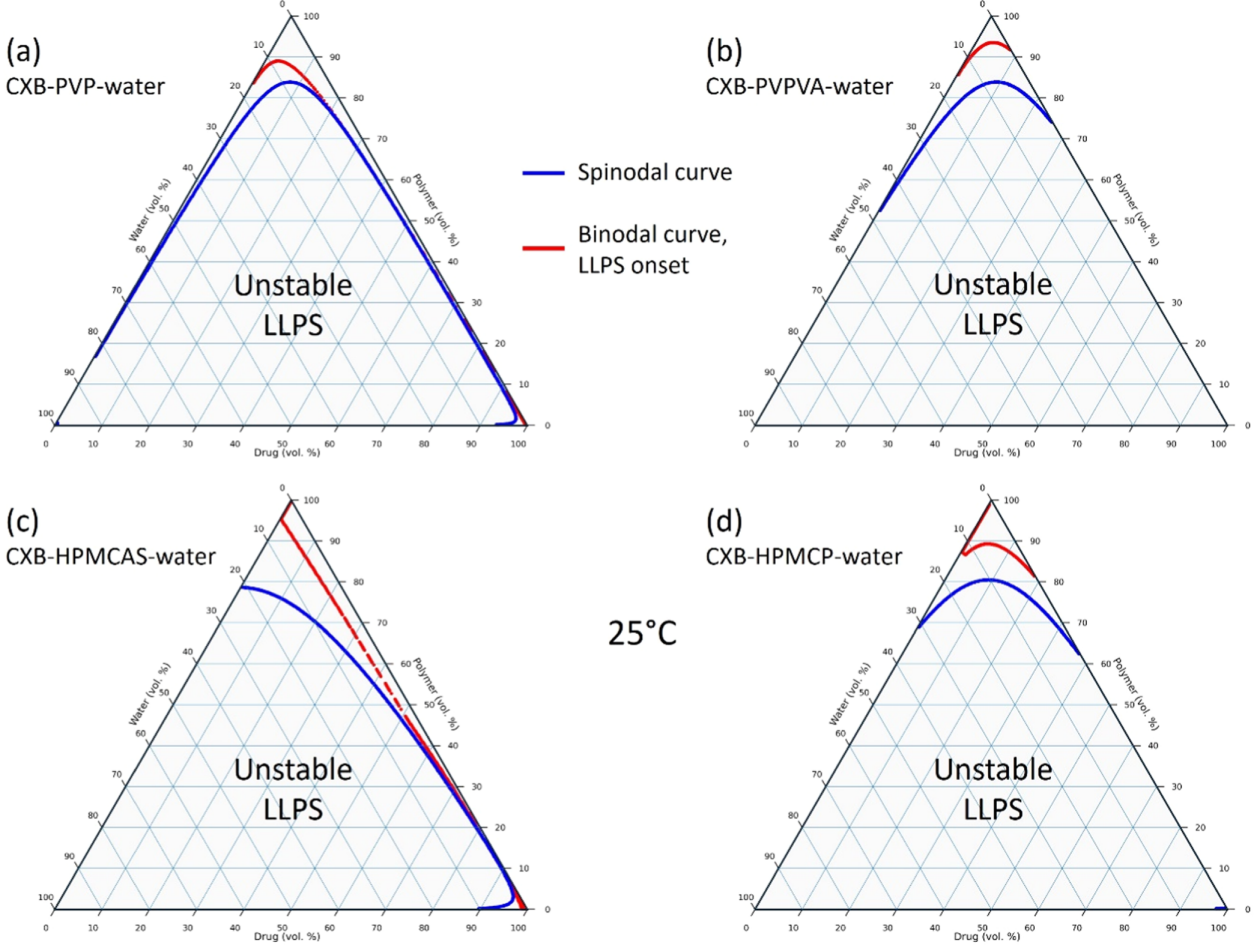
Ternary phase diagrams for systems of (a) CXB–PVP–water, (b) CXB–PVPVA–water, (c) CXB–HPMCAS–water, and (d) CXB–HPMCP–water constructed using classic Flory–Huggins interaction parameters at 25 °C; binodal and spinodal curves were marked in each diagram to highlight the phase boundaries with areas within the spinodal curve denoted as unstable LLPS regions.

CXB–polymer–water ternary phase diagrams for all four polymeric additives were constructed using previously established methods (Figure 2).^31^ The parameters such as molecular volume, density, solubility, and F–H interaction parameters are all provided in the Supporting Information. Within the diagram, the binodal and spinodal curves were plotted to highlight the phase behaviors of the CXB–polymer–water mixture. The ternary phase diagram illustrates the relevant compositions of the CXB supersaturated solutions with polymeric additives. However, given the limited resolution of the modeling tool used and the relatively small concentrations of the CXB in water, these ternary phase diagrams were not established using the existing set of F–H interaction parameters. Instead, four ternary phase diagrams were successfully constructed with lower values of the F–H interaction parameters (Figure S2).

Nevertheless, this approach illustrates the importance of polymeric additives for all four ternary systems at high polymer or water compositions. The locations of the LLPS boundary indicate the formation of droplets during this process. With these boundaries, the spinodal curves sit within the binodal curves, showing the limited local stability of the mixture in these compositions. The gaps between the spinodal and binodal curves are identified as the miscibility gap, highlighting the metastability nature of the LLPS. Within the miscibility gap, it is, therefore, most likely that the appearance of the CXB–polymer–water transient phases can be observed using various analytical techniques.^7^ The starting point of such an LLPS is understood to be very close to the binodal curve of the phase diagram; it is named the LLPS onset concentration in this work. The binodal curve defines the temperature and composition of the mixture at which phase separation is thermodynamically favorable. Following the positions of the binodal curves in all four systems in Figures 2 and S2, the identifiable areas are located at the three corners of the ternary phase diagram, e.g., the high polymer, high water, and high drug compositions. At these locations, the influences of the other two lower components may be illustrated by using the miscibility gaps. For example, high polymer and low drug composition areas indicate the possible phase behaviors of the drug–polymer ASD systems in water, as shown in the top areas in Figure 2. A homogeneous CXB–polymer ASD system may gradually move into the unstable LLPS when exposed to moisture during dissolution. Indeed, identifying the miscibility gaps for ternary systems has been widely used to develop formulations with nanoscale artifacts for many important commercial applications.^25,36^

Furthermore, it is observed that the shape of the miscibility gap is highly influenced by the polymeric additives in the CXB-water system, reflecting the differences in drug–polymer and water–polymer interaction parameters (Table S1). The wide miscibility gap from selecting appropriate polymers, e.g., HPMCAS, HPMCP, and PVPVA, is expected to increase the drug concentrations and the extent of supersaturation for CXB in the aqueous medium (LLPS). In comparison, the miscibility gap for the CXB–PVP–water system is relatively small if the values of interaction parameters are reduced proportionally (Table S1). The resulting ternary phase diagram appeared to have similar miscibility gaps at high water and low drug/polymer compositions, e.g., the supersaturated drug–water/polymer–water solutions. Although these constructed ternary phase diagrams are based on theoretical parameters derived using solubility parameters, the trend of such changes in the miscibility gaps reveals a reasonable outcome for the drug–polymer–water systems. With such a simple approach, one can quickly screen the polymeric additives for a given drug molecule for solubility enhancements and the likelihood of LLPS.^37^

In this work, the UV extinction measurement investigates the phase behaviors of supersaturated CXB solutions in the presence of various polymeric excipients. The supersaturated drug solution is achieved using the antisolvent/solvent-shifting method. Typically, the hydrophobic drug is dissolved in an organic solvent. The drug stock solution is then gradually added to the aqueous environment, where drugs reach the desirable supersaturation level until the LLPS occurs. This method hypothesizes that the limited fraction of organic solvent will not influence the phase behaviors of the system. The polar organic solvent in emulsion droplets will immediately disperse into the water. However, it is worth noting that due to the nature of this measurement, the appearance of the polymer–water phase separation can also cause UV extinction.

### 3.3 Factors Influencing the Detection of LLPS Onset Concentrations

Poorly water-soluble drug molecules exposed to an aqueous environment tend to precipitate through either the classical or nonclassical nucleation pathway. UV spectroscopy is one of the most commonly used techniques to characterize the drug-rich phases generated in supersaturated solutions.^38^ Typically, when electromagnetic radiation goes through a solution, the radiation may attenuate or become extinct due to absorption or scattering.^39,40^ The absorption occurs through the electrons of molecules in a solution, absorbing energy from radiation and expanding to a higher energy state. The scattering will be observed when insoluble particles are presented in the solution, where larger particles exhibit a higher level of scattering.^41,42^ In the drug–water systems, the particle scattering due to drug-rich phase generation was reported to lead to a spectrum baseline distortion, forming the foundation of this measurement.^43^ A high UV extinction is commonly observed at the high absorption regions of the drug molecule, ranging from 200 to 400 nm. PVP, PVPVA, and HPMCAS showed no absorption peak at wavelengths larger than 240 nm. HPMCP PBS solution had a notable peak at a wavelength of 292 nm. To minimize the UV extinction caused by polymer absorption, a wavelength of 360 nm was selected for all experiments. UV extinction spectra of CXB–PVPVA in PBS and MeOH solutions were verified at all relevant compositions with a 10–50 μg/mL drug concentration range in a 10 μg/mL interval (Figure S2). A clear step change in the relationship between drug concentrations and UV extinctions could be observed, indicating partial changes in the physical forms of the drug molecules within the solution. The occurrence of additional UV extinction was interpreted as the light scattering caused by the particles in high drug concentration samples, known as the Tyndall scattering.^43^ Therefore, the drug-rich phase onset point of the CXB–PVPVA–PBS ternary system could be estimated to be 28 μg/mL of CXB concentration.

**Figure 3:**
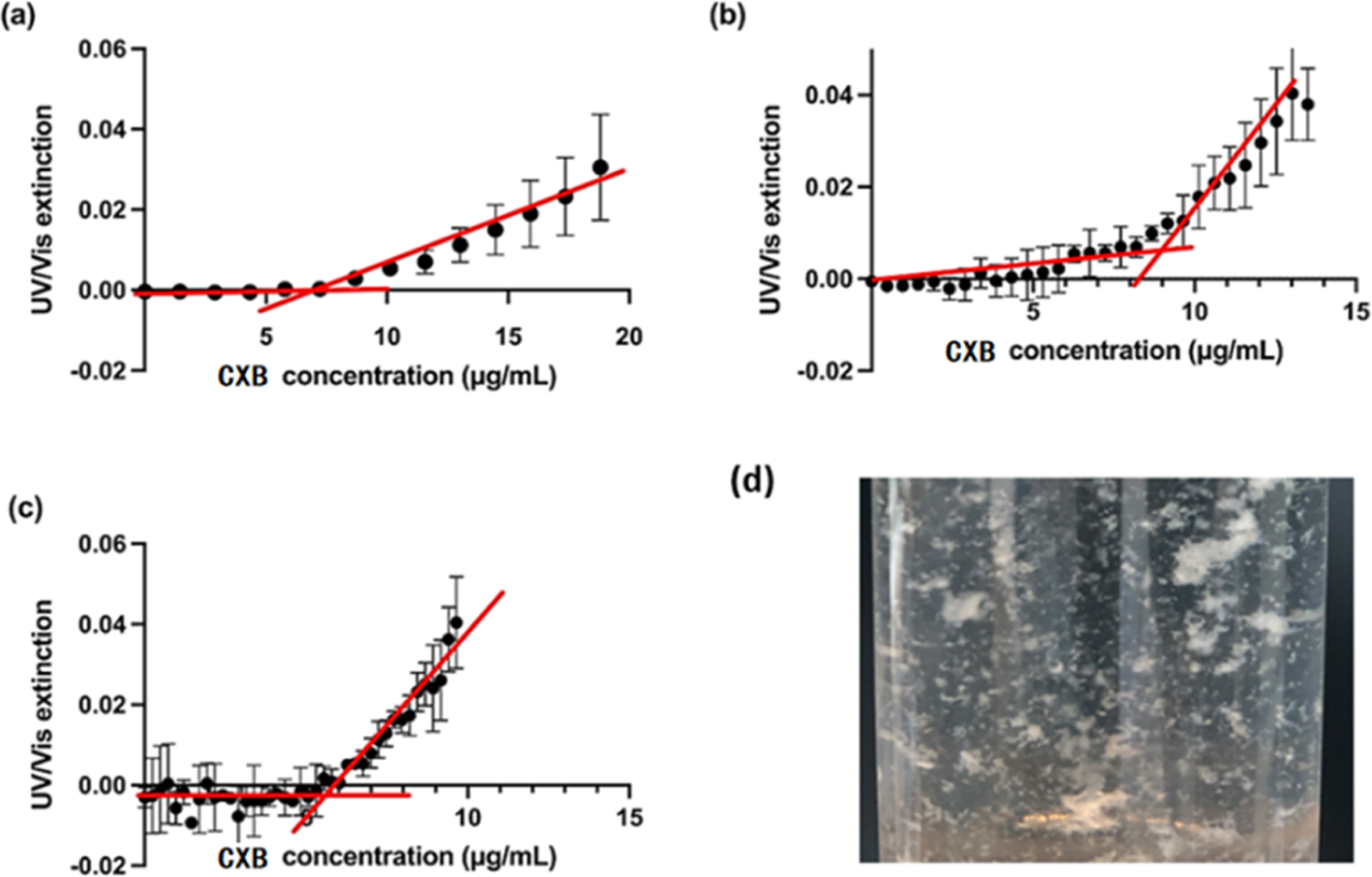
UV extinction of pure CXB solutions at the wavelength of 360 nm as a function of the drug concentration in the PBS buffer (pH = 7.4). The drug stock solution pumping rates were selected at (a) 3 mL/h, (b) 1 mL/h, and (c) 0.5 mL/h. (d) Precipitation of the CXB in PBS solution. Red lines represent regression curves.

First, without the presence of polymer, the UV extinction data of pure CXB solution as a function of CXB concentration at 37 °C are shown in Figure 3, with the drug stock solution pumping rates at 3, 1, and 0.5 mL/h. A wavelength of 360 nm was used to estimate the phase separation onset concentration of the CXB solution. To avoid interferences, the wavelength employed to determine the scattering intensity was far from the drug’s absorption wavelength range. The red lines were two least-squares regression curves fitted using data points at the initial stage and the subsequent appearance of supersaturated solutions with the increased CXB. The onset concentration of LLPS was derived from the intersection point of two red lines. The phase separation occurrence concentrations were determined as 7.75 ± 0.78, 8.58 ± 1.95, and 6.22 ± 1.07 μg/mL with the drug stock solution pumping rates of 3, 1, and 0.5 mL/h, respectively. No notable difference was observed with the drug stock solution pumping rate (*p >* 0.05). The drug concentration at the solution’s optical turbidity point has also been highly associated with the drug’s theoretical amorphous solubility. However, the experimentally detected phase separation concentration of pure CXB was remarkably lower than the theoretically calculated drug amorphous solubility. Using various theories, the CXB amorphous solubility was estimated to be 19–22.6 μg/mL.^44^ It was suggested that the fast recrystallization speed of the CXB has resulted in the formation of small crystalline drug particles before the LLPS. In this work, white precipitations could also be observed by the optical image of the pure CXB–PBS solution in a matter of minutes (Figure 3). Crystalline drug suspension in the solution may contribute to the overall UV spectrum scattering and lead to the step change at the slopes.^43^

The inline UV method can be affected by the appearance of all kinds of matters in the CXB titration experiment, such as the nanocrystalline, nanodroplet, amorphous nanoparticles or mixtures of all of the above.^3^ We also tried to pause the titration when the LLPS onset point was detected. The UV extinction value remained stable for at least 20 min, suggesting the number and size of the metastable particles were stable within the individual test.^30^ Additional experiments were also conducted to highlight the differences in UV extinction values caused by the crystalline CXB and LLPS (Figure S3). Seeding the CXB–PVP–water solution at a CXB concentration of 36 μg/mL with an additional 10% w/w crystalline CXB, a significant jump in the UV extinction was observed, which was higher than that caused by the LLPS. To further identify the compositions of the nanosized matter at the onset point of LLPS, the cryo-EM technique (Figure 6) and high-resolution TIRFM (with pyrene as the hydrophobic fluorescence probe, Supporting Information Videos 1–4) were used. The cryo-EM and TIRFM highlighted the appearance of spherical particles ranging from 40 nm to several micrometers. Particularly in the TIRFM with polarized filter videos, no significant birefringence was observed, indicating the possible aggregation of the CXB amorphous nanodroplet following the initial LLPS at much smaller sizes (Figure 6). The appearance of noncrystalline nano/microparticles with the CXB–polymer–water suspension highlighted the metastability nature of the mixture, thus validating that the main cause of the UV extinction is indeed attributed to the LLPS. It should be noted that several previous research articles presented the behaviors of LLPS for CXB in PBS with the predissolved polymeric matrices, such as PVP, PVPVA, and HPMCAS.^24,25,45,46^ These values suggest that under the experimental conditions described in this work (microfluidic pump and mixing), the onset of UV baseline change should be mainly attributed to LLPS in the CXB titration process (all experiments at various pumping rates were completed within 1 h). As mentioned, phase behaviors, e.g., LLPS in the drug–polymer–water ternary systems, were revealed to affect the solubility and permeability enhancement of ASD during oral administration.^25^ However, the polymer influence on the transient drug-rich phases is not fully understood. This section investigated various experimental conditions based on the onset point of LLPS for CXB–polymer–water ternary systems, including polymer types, polymer concentrations, drug–polymer interaction approaches, and drug–polymer mixing rates.

### 3.4 Effects of Polymer Types and Drug Stock Solution Pumping Rates

**Figure 4:**
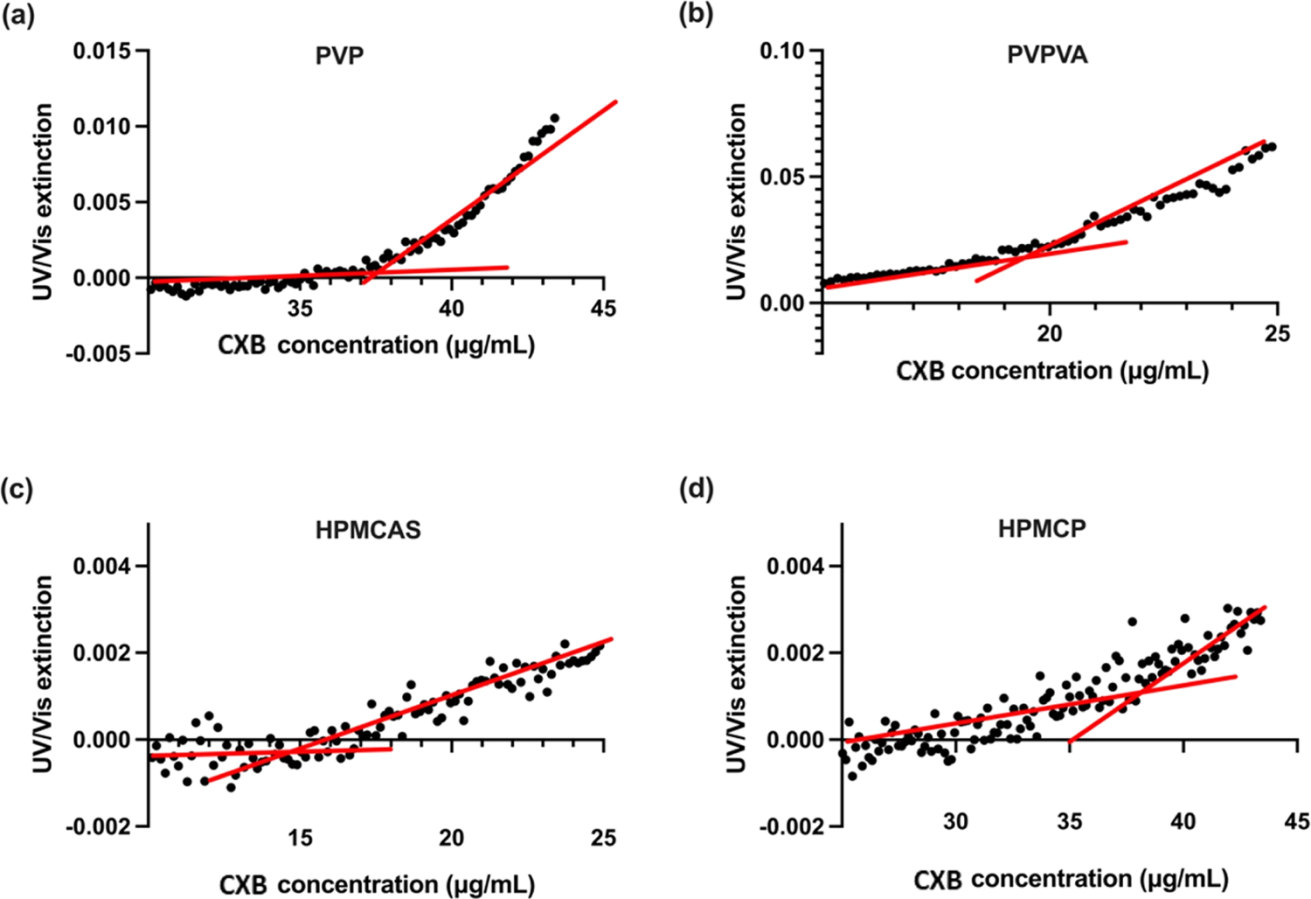
UV extinction profiles of CXB–polymer–water ternary systems as a function of CXB concentration (μg/mL), with the drug stock solution pumping rate of 3 mL/h. One mg/mL polymers of (a) PVP, (b) PVPVA, (c) HPMCAS, and (d) HPMCP were predissolved in the pH 7.4 PBS buffer at 37 °C. Red lines represent least-squares regression curves.

Given the usual low LLPS onset concentrations of most poorly water-soluble drugs in the aqueous medium, polymeric excipients are often predissolved to suppress the precipitations during the experiment. In this section, different polymers of PVP, PVPVA, HPMCAS, and HPMCP with a concentration of 1 mg/mL were predissolved in the PBS buffer to inhibit the precipitations of CXB. The phase behaviors of drug–polymer solutions were monitored using the same UV extinction method. The polymer type is a critical parameter that influences the LLPS onset concentration in a CXB–polymer–water ternary system, as the UV extinction profiles depicted in Figure 4 depend on the drug concentrations in the medium.

Compared with a pure CXB–PBS solution, it is clear that the LLPS onset concentrations have been altered for CXB when different polymeric materials are predissolved within the aqueous medium. The LLPS data varied in different polymer–water combinations. The LLPS onset concentrations for CXB ternary systems with predissolved PVP (Figure 4a) and HPMCP (Figure 4d) were recorded at 37.2 ± 0.77 and 37.6 ± 0.98 μg/mL, respectively. CXB ternary systems with polymers of PVPVA (Figure 4b) and HPMCAS (Figure 4c) exhibited lower LLPS onset concentrations, measured to be 18.0 ± 1.82 and 15.1 ± 1.33 μg/mL.

**Figure 5:**
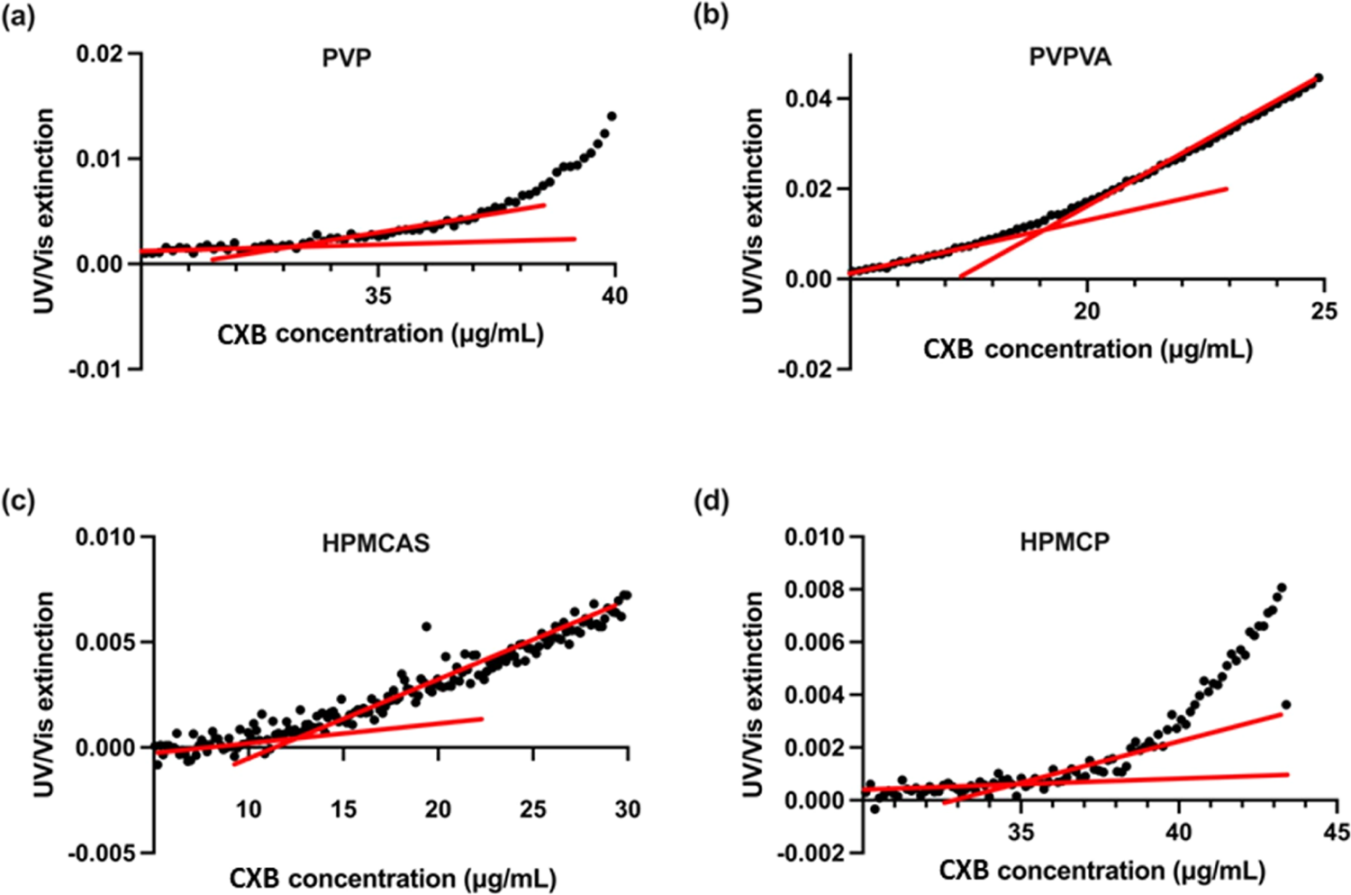
UV extinction profiles of CXB–polymer–water ternary systems as a function of CXB concentration (μg/mL), with the drug stock solution pumping rate of 0.5 mL/h. One mg/mL polymers of (a) PVP, (b) PVPVA, (c) HPMCAS, and (d) HPMCP were predissolved in the pH 7.4 PBS buffer at 37 °C. Red lines represent least-squares regression curves.

**Figure 6:**
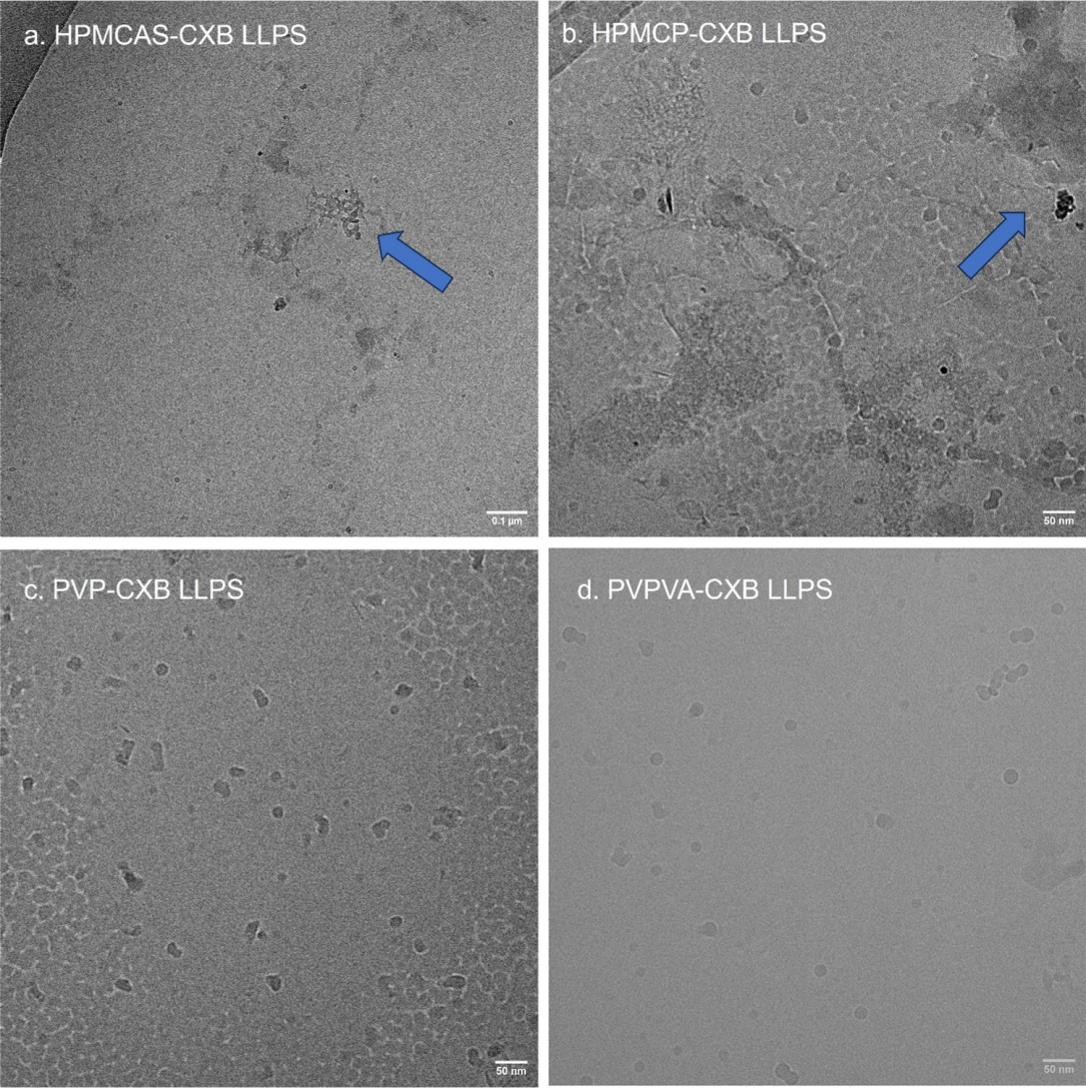
Micrographic representations of the CXB–polymer–PBS suspensions collected from the LLSP regions for (a) HPMCAS–CXB, (b) HPMCP–CXB, (c) PVP–CXB, and (d) PVPVA–CXB; blue arrows are pointing to the signs of agglomerations; scale bars are 100 nm for panel (a) and 50 nm for panels (b–d).

In a phase diagram, drug-rich phases were suggested to be generated in the metastable region. Drug-rich phases could be determined when these transient phases reach the local minimum energy position and are kinetically stable for a short period. The kinetic influence on the determination of the LLPS point was studied when the mixing rate of the stock solution and the PBS buffer was changed. This work suggested that the drug–polymer mixing rate is not a significant parameter of the LLPS onset concentration. Specifically, examples of UV extinction profiles of the CXB–polymer–water ternary system with drug stock solution pumping rates of 3 and 0.5 mL/h at the wavelength of 360 nm are shown in Figures 4 and 5; additional profiles of the UV extinction at a pumping rate of 1 mL/h are provided in Figure S3. To further verify the existence of metastable CXB–polymer–water transient phases, cryo-EM microscopic analysis was carried out for several selected samples after reaching LLPS onset points (Figure 6). Immediately after reaching the LLPS onset points, the liquids were drawn from the sample vials and rapidly frozen to achieve amorphous ice for cryo-EM. Round-shaped condensed matter with sizes of 20–80 nm was observed in all CXB–polymer–water systems. The amount of round-shaped condensed matter may indicate the CXB LLPS onset concentrations, where more spherical particles were observed in HPMCP and PVP-based systems than in HPMCAS and PVPVA mixtures. Furthermore, signs of agglomeration were also observed in HPMCAS and HPMCP-based CXB suspensions, reflecting the possible colloidal nature of these two polymeric matrices.^47^

**Figure 7:**
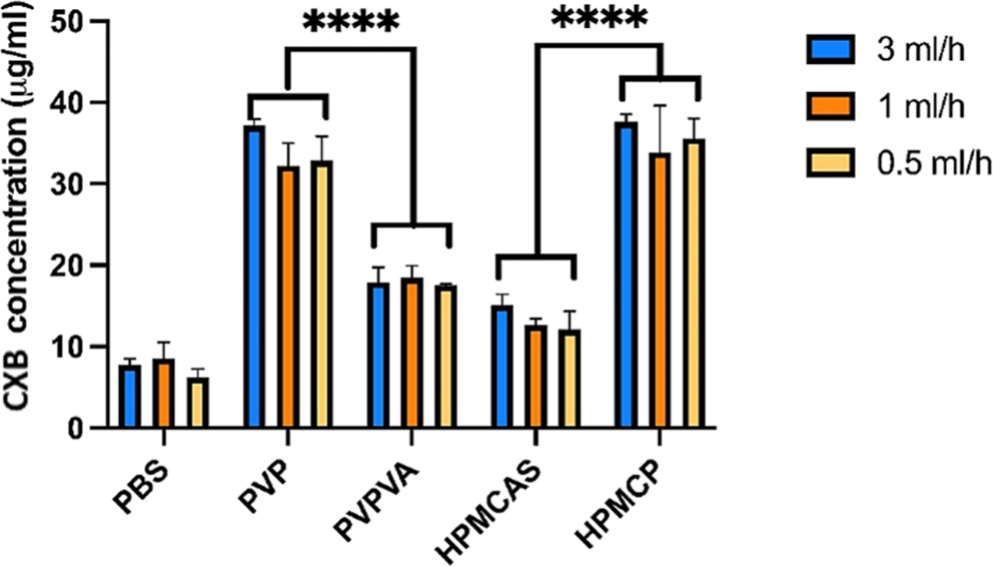
LLPS onset concentrations in the PBS solutions (pH 7.4) with or without predissolved polymers at 37 °C with various stock solution pumping speeds (*n* = 3). Blue, orange, and yellow bars represent the drug stock solution pumping rates of 3, 1, and 0.5 mL/h, respectively. Error bars derived from standard deviations. **** represent *p* < 0.0001.

LLPS onset concentrations in the PBS solution with or without polymers using various mixing rates are summarized in Table 1 and Figure 7. Values were calculated individually at different conditions. Error bars were derived from standard deviations of those values. Blue, orange, and yellow bars represent the drug stock solution (4 mg/mL) pumping rates of 3, 1, and 0.5 mL/h, respectively. 50 μg/mL CXB solutions were generated in 5, 15, and 30 min, respectively. CXB LLPS in PVP and HPMCP aqueous solutions exhibited significantly higher concentrations than those in PVPVA and HPMCAS solutions (*p* < 0.0001). No significant difference in the LLPS onset concentrations was observed when drug stock solution mixing rates were altered (two-way ANOVA, *p* > 0.05). The result suggested that the CXB LLPS point in the CXB–polymer–water ternary systems had a less kinetic influence within the first 30 min of the experiments. The presence of polymers influenced the LLPS onset concentrations remarkably by controlling the position of the binodal line. Samples with strong drug–polymer interactions were observed to undergo LLPS at high drug concentrations. For example, hydrogen bonding was identified within both CXB–PVP and CXB–PVPVA systems in the nonaqueous situation by ^1^H NMR spectra. At the mixing rate of 3 mL/h, the LLPS onset concentration for CXB at a 1 mg/mL PVP–PBS solution was approximately two times higher than that of the PVPVA solution, Table 1. A higher CXB LLPS onset value of the CXB–PVP system was interpreted by the stronger hydrogen bonding of the drug and polymer, evidenced by a more extensive chemical shift in ^1^H NMR spectra. Similarly, PVPVA and HPMCAS were reported to reduce the LLPS onset concentrations in other drug systems.^48^ It was suggested that the ibuprofen solubility was reduced in several polymer solutions, including the PVPVA, and that the bulk ibuprofen concentration was reduced with PVPVA. Miao et al. reported that the LLPS value of paclitaxel decreased from approximately 40–23 μg/mL with the HPMCAS (MF).

**Table 1:** CXB LLPS Onset Concentrations (μg/mL) in the PBS Solutions (pH 7.4) with or without 1 mg/mL Polymers of PVP, PVPVA, HPMCAS, and HPMCP at 37 °C^a^

		polymer types			
CXB induction rates	PBS	PVP	PVPVA	HPMCAS	HPMCP
3 mL/h	7.75 ± 0.78	37.2 ± 0.77	18.0 ± 1.82	15.1 ± 1.33	37.6 ± 0.98
1 mL/h	8.58 ± 1.95	32.3 ± 2.74	18.4 ± 1.50	12.7 ± 0.73	33.9 ± 5.72
0.5 mL/h	6.22 ± 1.07	32.9 ± 3.00	17.5 ± 0.20	±2.27	35.6 ± 2.40

a The drug stock solution pumping rates of 3, 1, and 0.5 mL/h were conducted in all polymer solutions (*n* = 3).

### 3.5 Role of Polymer Concentrations on the LLPS Onset

**Figure 8:**
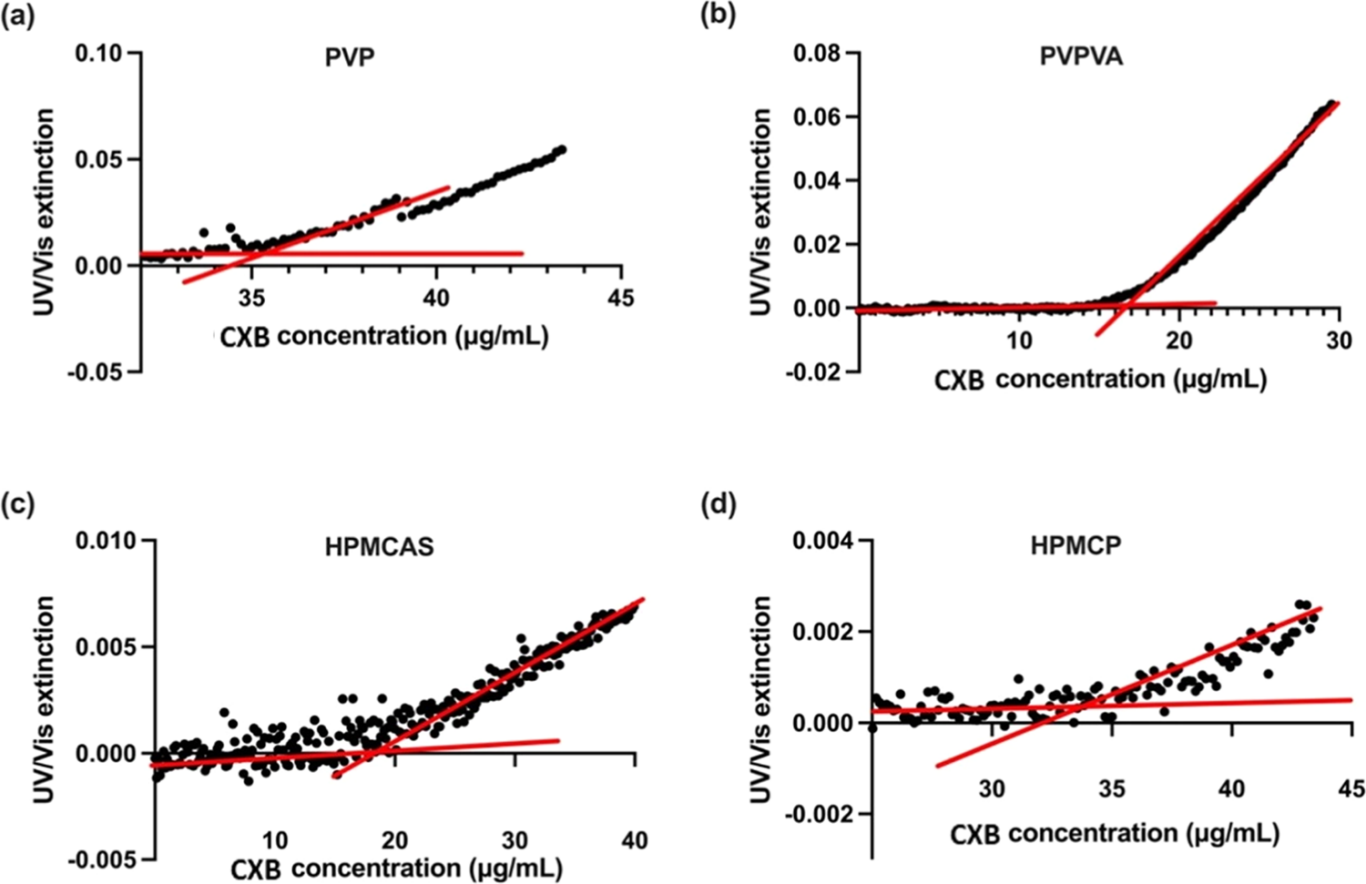
UV extinction profiles of CXB–polymer–water ternary systems as a function of CXB concentration (μg/mL), with the drug stock solution’s pumping speed of 1 mL/h. 500 μg/mL polymers of (a) PVP, (b) PVPVA, (c) HPMCAS, and (d) HPMCP were predissolved in the pH 7.4 PBS buffer at 37 °C. The red lines represent regression curves.

**Figure 9:**
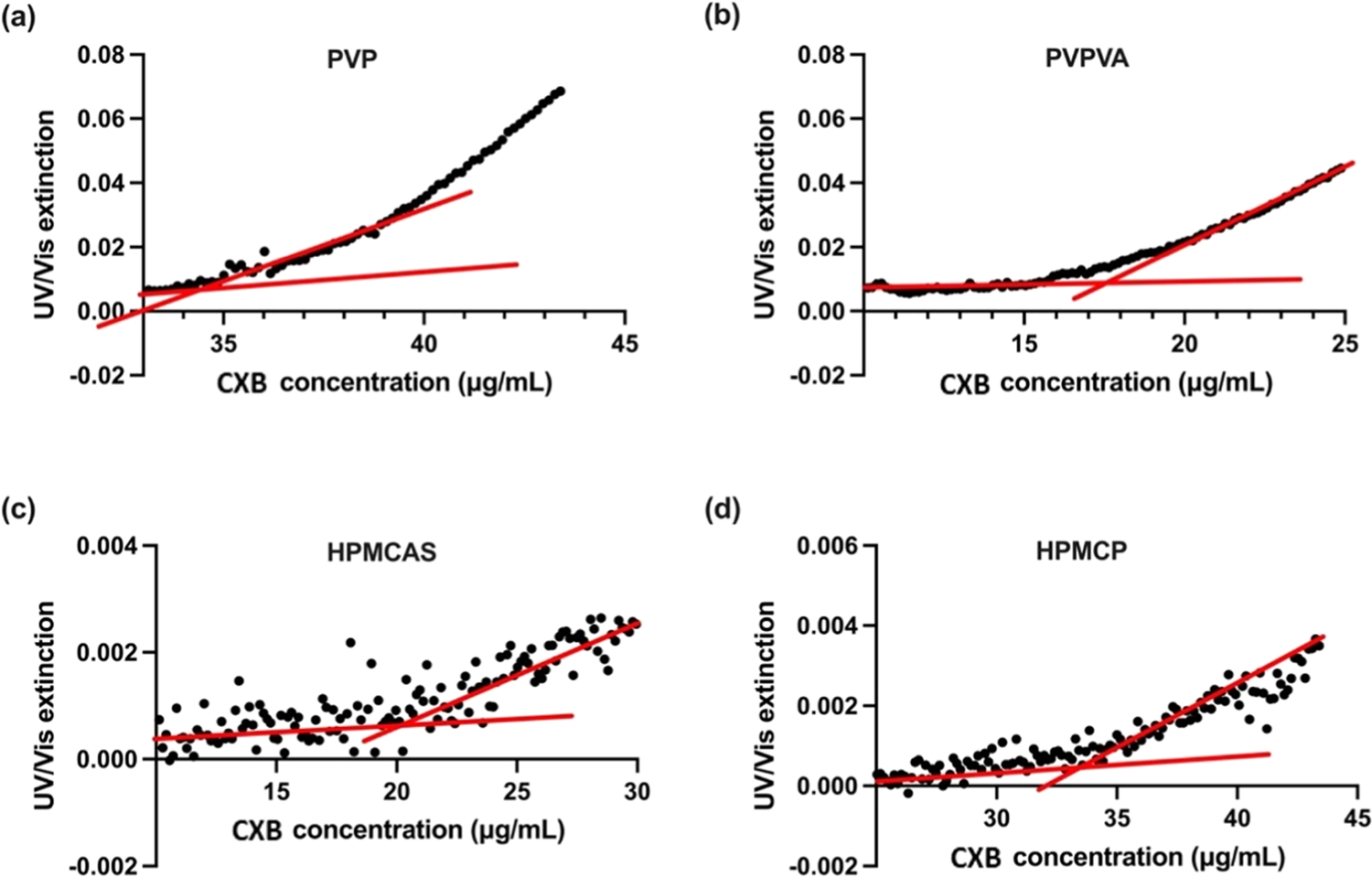
UV extinction profiles of CXB–polymer–water ternary systems as a function of CXB concentration (μg/mL), with the drug stock solution’s pumping speed of 1 mL/h. 0.1 mg/mL polymers of (a) PVP, (b) PVPVA, (c) HPMCAS, and (d) HPMCP were predissolved in the pH 7.4 PBS buffer at 37 °C. The red lines represent regression curves.

The drug–polymer composition has been commonly highlighted to influence the LLPS point in supersaturated drug-water solutions. This work determined the CXB LLPS onset concentrations of several CXB–polymer–water ternary systems with different polymer concentrations. UV extinction profiles as a function of drug concentration with the polymer concentrations at 500 and 100 μg/mL are shown in Figures 8 and 9. The LLPS onset concentrations at different systems were derived from the step change of regression curve slopes (red lines). Various LLPS onset concentrations observed for the CXB–polymer–water ternary system are summarized in Table 2 and Figure 9. Blue, orange, and yellow bars represent the polymer concentration at 1000, 500, and 100 μg/mL, respectively.

**Table 2:** LLPS Onset Concentrations (μg/mL) in the Predissolved PVP, PVPVA, HPMCAS, and HPMCP PBS Solutions (pH 7.4) with the Polymer Concentrations of 1000, 500, and 100 μg/mL at 37 °C (*n* = 3)^a^

	polymer types			
polymer concentration (μg/mL)	PVP	PVPVA	HPMCAS	HPMCP
1000	32.2 ± 2.74	18.4 ± 1.50	12.7 ± 5.72	33.9 ± 4.48
500	36.6 ± 0.64	16.5 ± 0.48	21.1 ± 2.27	33.9 ± 2.97
100	34.6 ± 1.48	17.3 ± 3.22	19.5 ± 4.29	32.4 ± 1.76

a The stock CXB solution induction rate was set at 1 mL/h.

**Figure 10:**
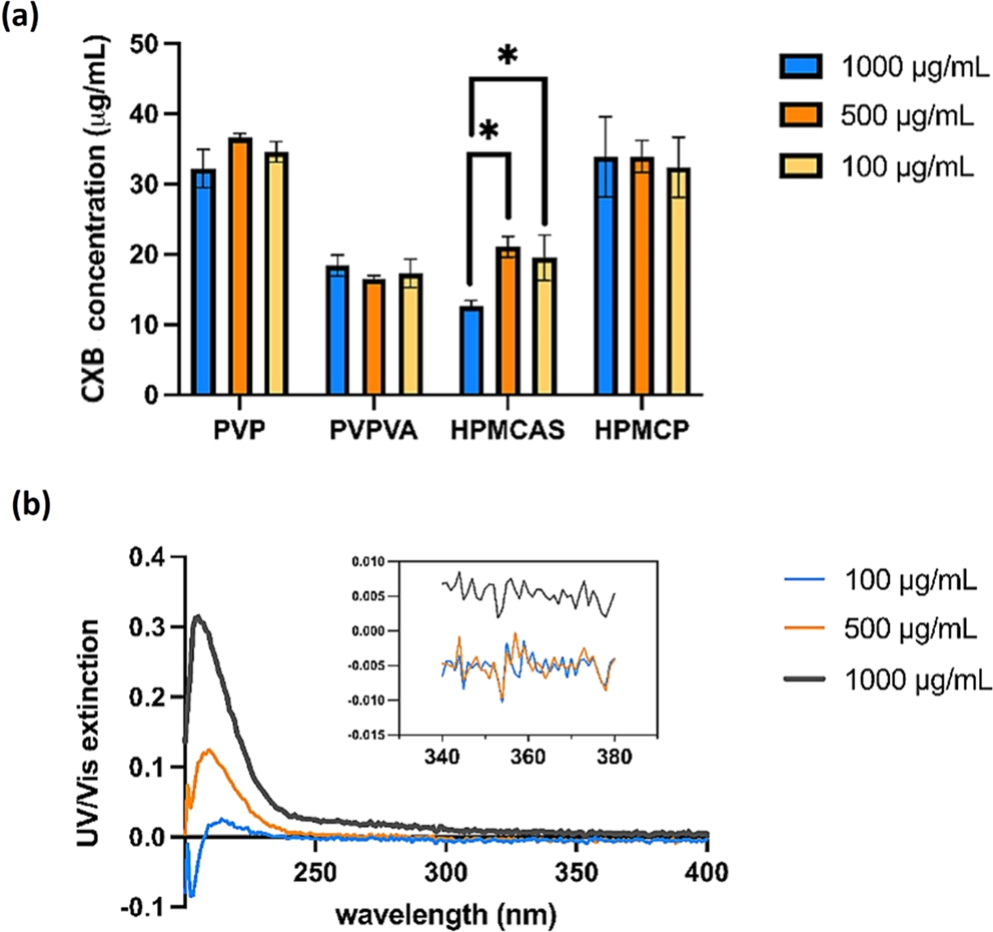
(a) LLPS onset concentrations of CXB in predissolved polymer solutions at 37 °C (*n* = 3). Blue, orange, and yellow bars represent the polymer concentration of 1000, 500, and 100 μg/mL, respectively—error bars derived from standard deviations. * represent *p* < 0.03. (b) The UV spectra of HPMCAS-MF PBS solution with polymer concentrations of 1000, 500, and 100 μg/mL, pH 7.4 at 37 °C.

In this case, CXB LLPS onset concentrations in the ternary systems were usually not altered when reducing the polymer concentration from 1000 μg/mL to 100 μg/mL (*p* > 0.05), as shown in Table 2 and Figure 10. However, the system with HPMCAS exhibited an abnormal LLPS concentration at a higher polymer concentration. The CXB LLPS concentration of the solution with the HPMCAS concentration of 1000 μg/mL was 12.7 ± 5.72 μg/mL. This value increased to 21.1 ± 2.27 μg/mL when the polymer concentration decreased to 500 μg/mL and then remained constant between 500 and 100 μg/mL.

The UV spectra of HPMCAS PBS solution (pH = 7.4) with a serial of polymer concentrations at 37 °C are illustrated in Figure 10b, where gray, orange, and blue curves represent HPMCAS concentrations of 1000, 500, and 100 μg/mL, respectively. Scattering was the sole factor contributing to the overall extinction at the LLPS determination wavelength (360 nm). It should be noted that the scattering was already observed in the HPMCAS solution at a concentration of 1000 μg/mL without the addition of CXB. This was due to the HPMCAS aggregated upon high polymer concentrations in the PBS solution (37 °C), where HPMCAS–PBS demixing occurred even without drug molecules (raised baseline in Figure 10b). Similar observations on the nature of colloid formation for HPMCAS at high concentrations have already been reported in the literature.^30,49^ To maintain the consistency of the experimental conditions among all polymeric carriers, the influence of HPMCAS aggregation was blanked out from the UV extinction before addition of the CXB stock solution. However, the results suggested that only approximately 12.7 μg/mL CXB was required to disrupt the existing HPMCAS aggregations in the PBS solution, resulting in a phase-separated CXB–HPMCAS colloid suspension in the PBS.

### 3.6 Influence of Preformed Drug–Polymer Interaction in Stock CXB Solution

The LLPS concept hypothesizes that the complexity of the free energy landscape can alter the dynamics of the resulting transient phase.^50^ In this case, the rate of mixing of CXB with water and the presence of polymeric carriers should be expected to alter the resulting LLPS onset point. The Flory–Huggins model is an important theoretical approach for estimating the phase boundaries in polymer-relevant solutions, which models the interaction of the components within a lattice theory.^51,52^ The entropic contribution to the free energy landscape of the system is determined by enumerating the distinct configurations of molecules and polymers within the lattice. In contrast, according to a regular solution theory, the enthalpic contribution arises from the paired interaction energies between the components.^53^ The component interaction is a dominating parameter influencing the free energy landscape and the LLPS. Previous sections studied the weaker interaction between the drug and various polymers, which can result in a lower LLPS onset concentration. Such alteration of drug–polymer interaction can also be complicated by moving the interaction from a nonaqueous state to an aqueous state. Chen et al. suggested that the drug–polymer intermolecular interaction strength in a nonaqueous environment may be weaker than in an aqueous solution.^54^ Marsac et al. found the hydrogen bonding between felodipine and PVP will be disrupted with the introduction of water.^55^

**Figure 11:**
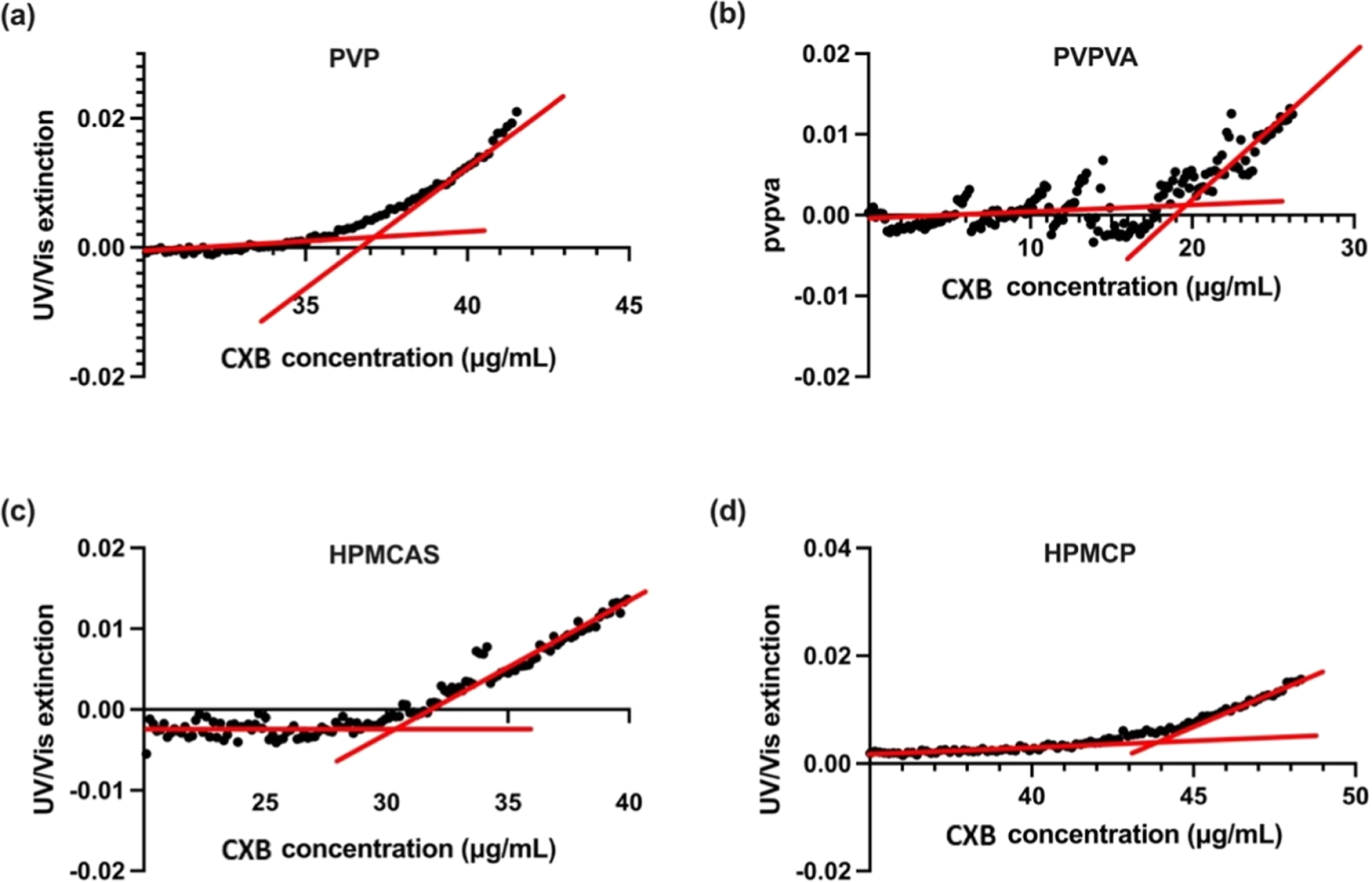
UV extinction profiles of CXB–polymer–water ternary systems as a function of CXB concentration (μg/mL), with the drug stock solution’s pumping speed of 1 mL/h. 0.5 mg/mL polymers of (a) PVP, (b) PVPVA, (c) HPMCAS, and (d) HPMCP were codissolved with drugs in the MeOH solution. Red lines represent regression curves.

Fundamentally, the experimental approach to obtain the LLPS of a small molecule drug in water is via solvent shift, where a drug-organic solvent solution is gradually added into a polymer–water solution. Quick diffusion of the organic solvent in water results in phase separation of the drug solution due to the poor water solubility. In this situation, the drug–polymer interaction is expected to form a competitive relationship with the water–polymer interactions. In comparison, the LLPS of the drug–polymer–water bond can also be obtained by adding a drug–polymer organic solvent solution into the water medium. However, in this case, drug–polymer interaction is expected to form in the organic solvent first and then be disrupted after mixing with water. This type of drug–polymer interaction is perhaps closer to the drug–polymer interactions formed within traditional amorphous solid dispersions, providing the relevance of this experimental approach for LLPS detection. To further investigate the impacts of the preformed drug–polymer interactions on the LLPS onset concentration in PBS media, organic solutions of drug–polymer systems were first prepared (codissolving method) (Table 3). In this approach, polymers and the CXB were codissolved in the MeOH, with a weight ratio of 2:1. 50 μg/mL of CXB and 100 μg/mL of polymers were expected at the end of the experiment. The stock solution mixing rate was set to 1 mL/h by the two methods. The influence of the polymer concentration on the LLPS onset concentration was negligible in this section due to the absence of a marked impact at low polymer concentrations. Extinction profiles of the CXB–polymer–water ternary system with 100 μg/mL of polymers introduced through the codissolving approach are depicted in Figure 11. LLPS onset concentrations of the ternary system were calculated by using the intersection point of regression curves.

**Table 3:** LLPS Onset Concentrations (μg/mL) of Solutions with Polymers Predissolved into the PBS or Codissolved with the CXB in the Drug Stock Solution at 37 °C (*n* = 3)^a^

	polymer types			
different mixing approaches	PVP	PVPVA	HPMCAS	HPMCP
predissolving	34.6 ± 1.48	17.3 ± 2.04	19.5 ± 3.22	32.4 ± 4.29
codissolving	37.5 ± 2.61	19.5 ± 2.17	31.2 ± 0.196	44.0 ± 4.40

a The pumping speed of the drug stock solution was 1 mL/h. The polymer solution was expected to be 100 μg/mL at the end of the experiment.

**Figure 12:**
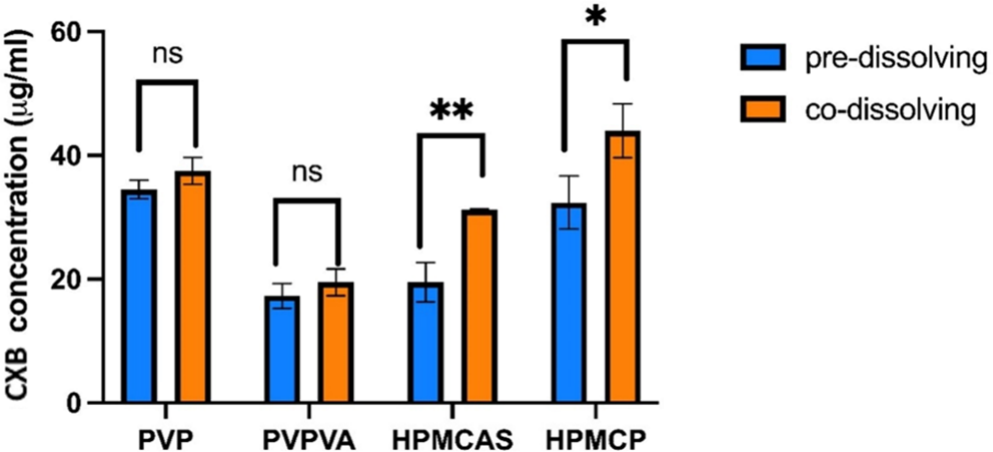
LLPS onset concentrations of solutions with polymers predissolved into the PBS or codissolved with the CXB in the stock solution. Blue bars represent the former polymer dissolving method, and orange bars represent the codissolved drug–polymer system before being added into the water. Error bars derived from standard deviations. ns, *, and ** represent not significant, *p* < 0.0332, and *p* < 0.0021, respectively.

LLPS onset concentrations of solutions with polymers predissolved in the PBS (pH 7.4) or codissolved with the CXB in the drug stock solution at 37 °C are shown in Table 3 and Figure 12. Blue bars represent the LLPS onset concentration of systems with predissolved polymers. Orange bars represent values calculated from systems when the CXB–polymer–MeOH stock solution was introduced into the pure PBS buffer. The CXB–polymer binary interaction was formed in a drug–polymer codissolving system before the organic droplet was dispersed into the water (Figure 1). The CXB concentrations at the LLPS point derived from the drug–polymer codissolving system were higher than those derived from the polymer–PBS predissolved method. In the case of a predissolved system, CXB needed to compete with water to interact with polymers. Notably, the LLPS onset concentration of systems in predissolved HPMCAS aqueous solution was significantly lower than the codissolved system, estimated to be 19.5 ± 3.22 and 31.2 ± 0.196 μg/mL, respectively. Similarly, the CXB LLPS onset concentration for the HPMCP codissolved system is indeed higher than the predissolved system, measured to be 44.0 ± 4.40 and 32.4 ± 4.29 μg/mL. The results suggested that the order of drug–polymer interaction is important in influencing the LLPS onset concentration of hydrophobic systems. However, this conclusion seems to not work in the hydrogen bonding-present systems (CXB–PVPVA and CXB–PVP systems). No notable difference has been observed in these samples (*p* > 0.03).

The drug–polymer interaction type may be another critical factor influencing the LLPS onset concentration. The hydrophobic interaction between CXB and polymers HPMCAS and HPMCP was encouraged via the codissolving method, where MeOH is the main medium. Hydrophobic interaction remains in the aqueous medium as MeOH diffuses into the water. The ternary system stays in one phase until a higher concentration of CXB is reached. Thus, the codissolving method can yield a much higher LLPS onset point in such systems than the predissolving method. In comparison, when hydrogen bonding is the dominant cause of drug–polymer interaction, it is far easier to disrupt by the water. Thus, orders of interactions between drugs and polymers (PVP, PVPVA) in an aqueous medium do not significantly affect the presence of the LLPS onset point of the system.^54^

### 3.7 Understanding the Dynamics of LLPS in a CXB–Polymer–Water Phase Diagram

**Figure 13:**
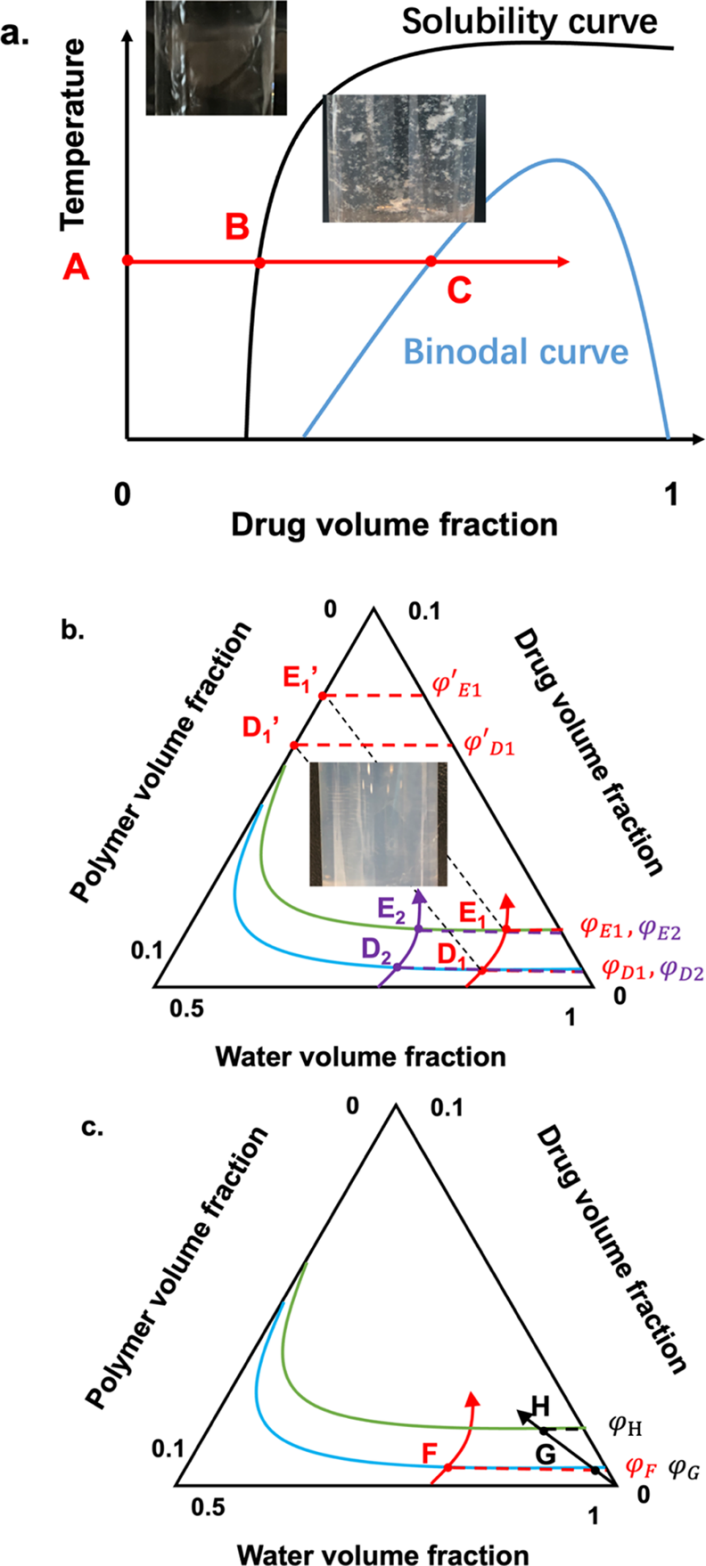
(a) Thermodynamic drug–water binary phase diagram. Solid black and blue lines represent the solubility and binodal lines, respectively. (b) Drug–polymer–water ternary system composition schematic phase diagram with various binodal curves (represented as the blue and green curves). Red and purple arrows illustrate the composition locus of systems with different starting polymer concentrations. (c) Drug–polymer–water ternary system composition schematic phase diagram. Red and black arrows represent the composition locus of systems in which polymers were predissolved in an aqueous solution and codissolved with a drug stock solution, respectively. The axis of the coordinates was adjusted.

Polymers significantly influenced the LLPS onset concentrations when using the codissolved CXB–polymer–MeOH approach. This fact demonstrated that understanding the drug–water binary system alone is inadequate for probing the drug release kinetics of ASD formulations. Without the presence of polymer additives, the drug precipitate was formed immediately without the observation of drug-rich phases. However, in most cases, the role of polymer additives and associated methodology is rather empirical for screening the polymer additives in a supersaturation study. The binary composition–temperature phase diagram perhaps helps us to understand the LLPS onset point while considering the polymer additives. Given a scenario of the drug concentration within the aqueous medium being any point between the solubility line and binodal line (Figure 13a), it is inevitable for the system to lower its energy by reducing the drug concentration in solution, moving toward point B. For the drug concentration to successfully move toward point C, polymer additives have been used to improve the kinetics stability of the drug–water binary system.^56,57^

In the case of HPMCAS as the predissolved polymeric additive in an aqueous medium, the addition of CXB effectively introduced the LLPS of the HPMCAS–water binary system at relatively low concentrations (<20 μg/mL CXB in water). It has also been repeatedly suggested that a strong drug–polymer interaction can promote a significant increase of drug solubility in aqueous solutions before reaching the LLPS onset point. To better describe these differences and highlight the role of polymeric additives in enhancing the drug’s solubility in water, a drug–polymer–water ternary phase diagram should be implemented as a routine approach (Figure 13b). Due to the limited drug and polymer concentration in aqueous solution, the axis of coordinate was adjusted to highlight the region of interest with the binodal curve (LLPS onset points); volume fraction scales between 0 and 0.1 for drug, 0–0.1 for polymer, and 0.5 and 1 for water.

Typically, drug–polymer systems with a strong interaction have a smaller binodal region and vice versa.^58^ The blue and green curves represent the binodal lines of the CXB–PVPVA and CXB–PVP systems, where the LLPS occurred at points *D*_1_ and *E*_1_, respectively. The ternary phase diagram estimated that the drug volume fraction at the LLPS onset point of the system with a weak drug–polymer interaction was lower than that of a system with a strong interaction (φ_*D*_1__ < φ_*E*_1__). A higher apparent drug volume fraction can be reached in an aqueous solution with a system that has a stronger CXB–polymer interaction. As we observed in this study, the polymer concentration did not influence the LLPS onset point. Purple and red arrows represent the LLPS routes with the different predissolved polymer concentrations. Two arrows intersected with the blue line at points *D*_1_ and *D*_2_ and the green line at points *E*_1_ and *E*_2_. For a given system, drug weight fractions of the drug-lean phases were very close to each other when altering the polymer concentration, where φ_*D*_1__ ≈ φ_*D*_2__, φ_*E*_1__ ≈ φ_*E*_2__. The shape of the curves in the phase diagram suggested that the effects of polymer concentrations within the system may not lead to significant changes in the drug concentration. In terms of the ASD dissolution, this shape of the binodal line suggests that the drug–polymer ratio of ASD may not potentially impact the LLPS onset concentration, thus limiting the solubility enhancement. A similar observation has also been reported in the literature in which the LLPS onset concentration of paclitaxel was not changed when increasing the HPMCAS concentration from 32 to 450 μg/mL.^48^

Drug–polymer interaction approaches also play an important role in LLPS. For systems with the water-resistant hydrophobic interaction, i.e., CXB–HPMCAS and CXB–HPMCP, it has been found that the determined CXB LLPS onset concentration from the codissolving approach was higher than that of the predissolving approach. However, for systems formed with water-sensitive hydrogen bonding, i.e., CXB–PVP and CXB–PVPVA, the LLPS onset concentrations were not significantly altered by the two different mixing approaches. Such a phenomenon was demonstrated in the ternary phase diagram, as illustrated in Figure 13c, where red and black arrows represented predissolving and codissolving mixing approaches. For the codissolving scheme, hydrophobic interactions between CXB and HPMCP or HPMCAS were revealed to form in the methanol, resulting in a smaller binodal region. These systems will separate at point *H* with a drug volume fraction of φ_*H*_. For the predissolving scheme, similar hydrophobic interaction was harder to form in the aqueous solution, and the LLPS can be estimated at point *F* and lead to a smaller drug volume fraction of φ_*F*_ (φ_*F*_ < φ_*H*_). In comparison, for a hydrogen bonding dominant system (CXB–PVP/PVPVA), the drug–polymer interaction was disrupted by water, irrespective of the different mixing approaches, resulting in a similar LLPS onset concentration (φ_*G*_ ≈ φ_*F*_).

In the current ASD design and development framework, the polymer property was revealed as a critical factor due to its contributions to miscibility, stability, and drug release performance.^59−63^ This work clarified that the presence of a polymer could also alter the dynamics of LLPS and the maximum achievable free drug concentration. In a standard dissolution study, the apparent solubility/concentration is always determined to assess the drug release performance. However, the concentration of the free drug without forming a complex with excipients was revealed to be the real driving force for improving drug absorption.^64^ Polymers that strongly interact with the drug will increase the LLPS onset concentration and the maximum achievable free drug concentration. When the drug concentration subsequently exceeds the LLPS onset concentration, the drug-rich phases are expected to reserve excess drugs, further facilitating drug absorption through the membrane.^47^

## 4 Conclusions

The LLPS onset point is a critical parameter that inherently indicates the maximum free drug concentration and generation of the drug-rich phase in a supersaturated drug solution. In previous works, the LLPS and generation of the drug-rich phase were understood using the drug–water binary phase diagram. It is unclear what the role of polymers played in generating drug-rich phases. This work systematically evaluated CXB drug-rich phases in PBS solutions combined with PVP, PVPVA, HPMCAS, and HPMCP polymers using the solvent/shifting method. The strength of the drug–polymer interaction and the orders of drug–polymer interactions were revealed to alter the dynamics of LLPS. However, parameters like the polymer concentration and the mixing rate of drug and polymer were found to be less significant for the LLPS onset concentration of CXB solutions. The general phase diagram of the drug–polymer–water ternary system was utilized to understand the LLPS onset points determined from various CXB–polymer supersaturated solutions. This study highlighted the importance of polymers in generating the metastable drug-rich transient phases by implementing LLPS of the ternary phase diagram.

## Supporting Information Available

Additional information on the selection of UV/vis wavelength for detecting the LLPS onset points, the parameters for construction of the ternary phase diagrams, and videos of the CXB–polymer–water LLPS are provided in the supporting information (PDF)

## References

[ref1] Bhujbal Sv.; Mitra B.; Jain U.; Gong Y.; Agrawal A.; Karki S.; Taylor L. S.; Kumar S.; Tony Zhou Q. Pharmaceutical Amorphous Solid Dispersion: A Review of Manufacturing Strategies. Acta Pharm. Sin. B 2021, 2505–2536. 10.1016/j.apsb.2021.05.014. 34522596 PMC8424289

[ref2] Yang R.; Zhang G. G. Z.; Zemlyanov D. Y.; Purohit H. S.; Taylor L. S. Release Mechanisms of Amorphous Solid Dispersions: Role of Drug-Polymer Phase Separation and Morphology. J. Pharm. Sci. 2023, 112 (1), 304–317. 10.1016/j.xphs.2022.10.021. 36306863

[ref3] Qian K.; Stella L.; Jones D. S.; Andrews G. P.; Du H.; Tian Y. Drug-Rich Phases Induced by Amorphous Solid Dispersion: Arbitrary or Intentional Goal in Oral Drug Delivery?. Phamaceutics 2021, 13 (6), 889 10.3390/pharmaceutics13060889. PMC8232734 34203969

[ref4] Xu Y.; Tijssen K. C. H.; Bomans P. H. H.; Akiva A.; Friedrich H.; Kentgens A. P. M.; Sommerdijk N. A. J. M. Microscopic Structure of the Polymer-Induced Liquid Precursor for Calcium Carbonate. Nat. Commun. 2018, 9 (1), 2582 10.1038/s41467-018-05006-w. 29968713 PMC6030133

[ref5] Hyman A. A.; Weber C. A.; Jülicher F. Liquid-Liquid Phase Separation in Biology. Annu. Rev. Cell Dev. Biol. 2014, 30 (1), 39–58. 10.1146/annurev-cellbio-100913-013325. 25288112

[ref6] Wang S.; Dai T.; Qin Z.; Pan T.; Chu F.; Lou L.; Zhang L.; Yang B.; Huang H.; Lu H.; Zhou F. Targeting Liquid–Liquid Phase Separation of SARS-CoV-2 Nucleocapsid Protein Promotes Innate Antiviral Immunity by Elevating MAVS Activity. Nat. Cell Biol. 2021, 23 (7), 718–732. 10.1038/s41556-021-00710-0. 34239064

[ref7] Ianiro A.; Wu H.; van Rijt M. M. J.; Vena M. P.; Keizer A. D. A.; Esteves A. C. C.; Tuinier R.; Friedrich H.; Sommerdijk N. A. J. M.; Patterson J. P. Liquid–Liquid Phase Separation during Amphiphilic Self-Assembly. Nat. Chem. 2019, 11 (4), 320–328. 10.1038/s41557-019-0210-4. 30778139

[ref8] de Yoreo J. A Perspective on Multistep Pathways of Nucleation. ACS Symp. Ser. 2020, 1358, 1–17. 10.1021/bk-2020-1358.ch001.

[ref9] Qin D.; He Z.; Li P.; Zhang S. Liquid-Liquid Phase Separation in Nucleation Process of Biomineralization. Front Chem. 2022, 10 (February), 1–11. 10.3389/fchem.2022.834503. PMC8854647 35186885

[ref10] Freedman M. A. Liquid-Liquid Phase Separation in Supermicrometer and Submicrometer Aerosol Particles. Acc. Chem. Res. 2020, 53 (6), 1102–1110. 10.1021/acs.accounts.0c00093. 32432453

[ref11] Gebauer D.; Raiteri P.; Gale J. D.; Cölfen H. On Classical and Non-Classical Views on Nucleation. Am. J. Sci. 2018, 318 (9), 969 10.2475/09.2018.05.

[ref12] Demichelis R.; Raiteri P.; Gale J. D.; Quigley D.; Gebauer D. Stable Prenucleation Mineral Clusters Are Liquid-like Ionic Polymers. Nat. Commun. 2011, 2, 590 10.1038/ncomms1604. 22186886 PMC3247826

[ref13] Avaro J. T.; Wolf S. L. P.; Hauser K.; Gebauer D. Stable Prenucleation Calcium Carbonate Clusters Define Liquid–Liquid Phase Separation. Angew. Chem., Int. Ed. 2020, 59 (15), 6155–6159. 10.1002/anie.201915350. PMC7187218 31943581

[ref14] Gebauer D.; Cölfen H. Prenucleation Clusters and Non-Classical Nucleation. Nano Today 2011, 6, 564–584. 10.1016/j.nantod.2011.10.005.

[ref15] Zahn D. Thermodynamics and Kinetics of Prenucleation Clusters, Classical and Non-Classical Nucleation. ChemPhysChem 2015, 16 (10), 2069–2075. 10.1002/cphc.201500231. 25914369 PMC4529657

[ref16] Sear R. P. The Non-Classical Nucleation of Crystals: Microscopic Mechanisms and Applications to Molecular Crystals, Ice and Calcium Carbonate. Int. Mater. Rev. 2012, 57 (6), 328–356. 10.1179/1743280411Y.0000000015.

[ref17] Karthika S.; Radhakrishnan T. K.; Kalaichelvi P. A Review of Classical and Nonclassical Nucleation Theories. Cryst. Growth Des. 2016, 16 (11), 6663–6681. 10.1021/acs.cgd.6b00794.

[ref18] Erdemir D.; Lee A. Y.; Myerson A. S. Nucleation of Crystals from Solution: Classical and Two-Step Models. Acc. Chem. Res. 2009, 42 (5), 621–629. 10.1021/ar800217x. 19402623

[ref19] Vekilov P. G. Nucleation. Cryst. Growth Des. 2010, 10 (12), 5007–5019. 10.1021/cg1011633. PMC2995260 21132117

[ref20] Martin E. W.; Harmon T. S.; Hopkins J. B.; Chakravarthy S.; Incicco J. J.; Schuck P.; Soranno A.; Mittag T. A Multi-Step Nucleation Process Determines the Kinetics of Prion-like Domain Phase Separation. Nat. Commun. 2021, 12 (1), 4513 10.1038/s41467-021-24727-z. 34301955 PMC8302766

[ref21] Altena F. W.; Smolders C. A. Calculation of Liquid-Liquid Phase Separation in a Ternary System of a Polymer in a Mixture of a Solvent and a Nonsolvent. Macromolecules 1982, 15 (6), 1491–1497. 10.1021/ma00234a008.

[ref22] Romay M.; Diban N.; Urtiaga A. Thermodynamic Modeling and Validation of the Temperature Influence in Ternary Phase Polymer Systems. Polymers 2021, 13 (5), 678 10.3390/polym13050678. 33668209 PMC7956791

[ref23] Kahrs C.; Gühlstorf T.; Schwellenbach J. Influences of Different Preparation Variables on Polymeric Membrane Formation via Nonsolvent Induced Phase Separation. J. Appl. Polym. Sci. 2020, 137 (28), 1–19. 10.1002/app.48852.

[ref24] Xie T.; Taylor L. S. Improved Release of Celecoxib from High Drug Loading Amorphous Solid Dispersions Formulated with Polyacrylic Acid and Cellulose Derivatives. Mol. Pharm. 2016, 13 (3), 873 10.1021/acs.molpharmaceut.5b00798. 26791934

[ref25] Andrews G. P.; Qian K.; Jacobs E.; Jones D. S.; Tian Y. High Drug Loading Nanosized Amorphous Solid Dispersion (NASD) with Enhanced in Vitro Solubility and Permeability: Benchmarking Conventional ASD. Int. J. Pharm. 2023, 632, 122551 10.1016/j.ijpharm.2022.122551. 36581107

[ref26] Ilevbare G. A.; Taylor L. S. Liquid-Liquid Phase Separation in Highly Supersaturated Aqueous Solutions of Poorly Water-Soluble Drugs: Implications for Solubility Enhancing Formulations. Cryst. Growth Des. 2013, 13 (4), 1497–1509. 10.1021/cg301679h.

[ref27] Purohit H. S.; Taylor L. S. Phase Behavior of Ritonavir Amorphous Solid Dispersions during Hydration and Dissolution. Pharm. Res. 2017, 34 (12), 2842–2861. 10.1007/s11095-017-2265-5. 28956218

[ref28] Deac A.; Qi Q.; Indulkar A. S.; Purohit H. S.; Gao Y.; Zhang G. G. Z.; Taylor L. S. Dissolution Mechanisms of Amorphous Solid Dispersions: Role of Drug Load and Molecular Interactions. Mol. Pharm. 2023, 20 (1), 722 10.1021/acs.molpharmaceut.2c00892. 36545917

[ref29] Ueda K.; Higashi K.; Moribe K. Quantitative Analysis of Drug Supersaturation Region by Temperature-Variable Nuclear Magnetic Resonance Measurements, Part 1: Effects of Polymer and Drug Chiralities. Mol. Pharm. 2023, 20 (4), 1861 10.1021/acs.molpharmaceut.2c00924. 36939575

[ref30] Bristol A. N.; Lamm M. S.; Li Y. Impact of Hydroxypropyl Methylcellulose Acetate Succinate Critical Aggregation Concentration on Celecoxib Supersaturation. Mol. Pharm. 2021, 18 (12), 4299 10.1021/acs.molpharmaceut.1c00372. 34738825

[ref31] Tian Y.; Qian K.; Jacobs E.; Amstad E.; Jones D. S. D. S.; Stella L.; Andrews G. P. G. P. The Investigation of Flory – Huggins Interaction Parameters for Amorphous Solid Dispersion Across the Entire Temperature and Composition Range. Pharmaceutics 2019, 11 (8), 420 10.3390/pharmaceutics11080420. 31430958 PMC6722828

[ref32] Xie T.; Taylor L. S. Effect of Temperature and Moisture on the Physical Stability of Binary and Ternary Amorphous Solid Dispersions of Celecoxib. J. Pharm. Sci. 2017, 106 (1), 100–110. 10.1016/j.xphs.2016.06.017. 27476771

[ref33] Rask M. B.; Knopp M. M.; Olesen N. E.; Holm R.; Rades T. Influence of PVP/VA Copolymer Composition on Drug-Polymer Solubility. Eur. J. Pharm. Sci. 2016, 85, 10–17. 10.1016/j.ejps.2016.01.026. 26826280

[ref34] Cappello B.; di Maio C.; Iervolino M.; Miro A. Combined Effect of Hydroxypropyl Methylcellulose and Hydroxypropyl-β- Cyclodextrin on Physicochemical and Dissolution Properties of Celecoxib. J. Inclusion Phenom. Macrocyclic Chem. 2007, 59 (3–4), 237–244. 10.1007/s10847-007-9319-y.

[ref35] Ventura C. A.; Tommasini S.; Falcone A.; Giannone I.; Paolino D.; Sdrafkakis V.; Mondello M. R.; Puglisi G. Influence of Modified Cyclodextrins on Solubility and Percutaneous Absorption of Celecoxib through Human Skin. Int. J. Pharm. 2006, 314 (1), 37–45. 10.1016/j.ijpharm.2006.02.006. 16581211

[ref36] Sengupta R.; Chakraborty S.; Bandyopadhyay S.; Dasgupta S.; Mukhopadhyay R.; Auddy K.; Deuri A. S. A Short Review on Rubber/Clay Nanocomposites With Emphasis on Mechanical Properties. Engineering 2007, 47, 21–25. 10.1002/pen.

[ref37] Dohrn S.; Kyeremateng S. O.; Bochmann E.; Sobich E.; Wahl A.; Liepold B.; Sadowski G.; Degenhardt M. Thermodynamic Modeling of the Amorphous Solid Dispersion-Water Interfacial Layer and Its Impact on the Release Mechanism. Pharmaceutics 2023, 15 (5), 1539 10.3390/pharmaceutics15051539. 37242781 PMC10221441

[ref38] Tomaszewska E.; Soliwoda K.; Kadziola K.; Tkacz-szczesna B.; Celichowski G.; Cichomski M.; Szmaja W.; Grobelny J. Detection Limits of DLS and UV-Vis Spectroscopy in Characterization of Polydisperse Nanoparticles Colloids. J. Nanomater. 2013, 2013 10.1155/2013/313081.

[ref39] Bohren C. F.; Huffman D. R. Absorption and Scattering of Light by Small Particles; Wiley, 1983. 10.1088/0031-9112/35/3/025.

[ref40] H. C., Van de Hulst. Light Scattering by Small Particles; Dover Publications, Inc., New York, 1981.

[ref41] Eshel G.; Levy G. J.; Mingelgrin U.; Singer M. J. Critical Evaluation of the Use of Laser Diffraction for Particle-Size Distribution Analysis. Soil Sci. Soc. Am. J. 2004, 68 (3), 736–743. 10.2136/sssaj2004.7360.

[ref42] Carvalho P. M.; Felício M. R.; Santos N. C.; Gonçalves S.; Domingues M. M. Application of Light Scattering Techniques to Nanoparticle Characterization and Development. Front Chem. 2018, 6 (June), 237 10.3389/fchem.2018.00237. 29988578 PMC6026678

[ref43] van Eerdenbrugh B.; Alonzo D. E.; Taylor L. S. Influence of Particle Size on the Ultraviolet Spectrum of Particulate-Containing Solutions: Implications for in-Situ Concentration Monitoring Using UV/Vis Fiber-Optic Probes. Pharm. Res. 2011, 28 (7), 1643–1652. 10.1007/s11095-011-0399-4. 21374101

[ref44] Abu-diak A. O.; Jones S. D.; Andrews P. G. Understanding the Performance of Melt Extruded Poly Ethylene Oxide Bicalutamide Solid Dispersions Characterisation of Microstructual Properties Using Thermal Spectroscopic and Drug Release Methods. J. Pharm. Sci. 2012, 101 (1), 200–213. 10.1002/jps. 21905037

[ref45] Knopp M. M.; Nguyen J. H.; Mu H.; Langguth P.; Rades T.; Holm R. Influence of Copolymer Composition on In Vitro and In Vivo Performance of Celecoxib-PVP/VA Amorphous Solid Dispersions. AAPS Journal 2016, 18 (2), 416–423. 10.1208/s12248-016-9865-6. 26769250 PMC4779114

[ref46] Ha E. S.; Choo G. H.; Baek I. H.; Kim M. S. Formulation, Characterization, and in Vivo Evaluation of Celecoxib-PVP Solid Dispersion Nanoparticles Using Supercritical Antisolvent Process. Molecules 2014, 19 (12), 20325–20339. 10.3390/molecules191220325. 25486246 PMC6271652

[ref47] Stewart A. M.; Grass M. E.; Brodeur T. J.; Goodwin A. K.; Morgen M. M.; Friesen D. T.; Vodak D. T. Impact of Drug-Rich Colloids of Itraconazole and HPMCAS on Membrane Flux in Vitro and Oral Bioavailability in Rats. Mol. Pharmaceutics 2017, 14 (7), 2437–2449. 10.1021/acs.molpharmaceut.7b00338. 28591516

[ref48] Miao L.; Liang Y.; Pan W.; Gou J.; Yin T.; Zhang Y.; He H.; Tang X. Effect of Supersaturation on the Oral Bioavailability of Paclitaxel/Polymer Amorphous Solid Dispersion. Drug Delivery Transl. Res. 2019, 9 (1), 344–356. 10.1007/s13346-018-0582-9. 30187352

[ref49] Ueda K.; Taylor L. S. Polymer Type Impacts Amorphous Solubility and Drug-Rich Phase Colloidal Stability: A Mechanistic Study Using Nuclear Magnetic Resonance Spectroscopy. Mol. Pharmaceutics 2020, 17 (4), 1352–1362. 10.1021/acs.molpharmaceut.0c00061. 32097023

[ref50] De Yoreo J. J.; Gilbert P. U. P. A.; Sommerdijk N. A. J. M.; Penn R. L.; Whitelam S.; Joester D.; Zhang H.; Rimer J. D.; Navrotsky A.; Banfield J. F.; Wallace A. F.; Michel F. M.; Meldrum F. C.; Cölfen H.; Dove P. M. Crystallization by Particle Attachment in Synthetic, Biogenic, and Geologic Environments. Science 2015, 349, aaa6760 10.1126/science.aaa6760. 26228157

[ref51] Nozary S.; Modarress H.; Eliassi A. Cloud-Point Measurements for Salt + Poly(Ethylene Glycol) + Water Systems by Viscometry and Laser Beam Scattering Methods. J. Appl. Polym. Sci. 2003, 89 (7), 1983–1990. 10.1002/app.12450.

[ref52] Flory P. J. Principles of Polymer Chemistry; Cornell University Press: New York, 1953.

[ref53] Hildebrand J. H.; Wood S. E. The Derivation of Equations for Regular Solutions. J. Chem. Phys. 1933, 1 (12), 817 10.1063/1.1749250.

[ref54] Chen Y.; Pui Y.; Chen H.; Wang S.; Serno P.; Tonnis W.; Chen L.; Qian F. Polymer-Mediated Drug Supersaturation Controlled by Drug-Polymer Interactions Persisting in an Aqueous Environment. Mol. Pharmaceutics 2019, 16 (1), 205–213. 10.1021/acs.molpharmaceut.8b00947. 30452278

[ref55] Marsac P. J.; Rumondor A. C. F.; Nivens D. E.; Kestur U. S.; Lia S.; Taylor L. S. Effect of Temperature and Moisture on the Miscibility of Amorphous Dispersions of Felodipine and Poly(Vinyl Pyrrolidone). J. Pharm. Sci. 2010, 99 (1), 169–185. 10.1002/jps.21809. 19492305

[ref56] Hate S. S.; Reutzel-Edens S. M.; Taylor L. S. Insight into Amorphous Solid Dispersion Performance by Coupled Dissolution and Membrane Mass Transfer Measurements. Mol. Pharmaceutics 2019, 16 (1), 448–461. 10.1021/acs.molpharmaceut.8b01117. 30521350

[ref57] Raina S. A.; Zhang G. G. Z.; Alonzo D. E.; Wu J.; Zhu D.; Catron N. D.; Gao Y.; Taylor L. S. Enhancements and Limits in Drug Membrane Transport Using Supersaturated Solutions of Poorly Water Soluble Drugs. J. Pharm. Sci. 2014, 103 (9), 2736–2748. 10.1002/jps.23826. 24382592

[ref58] Baghel S.; Cathcart H.; O’Reilly N. J. Theoretical and Experimental Investigation of Drug-Polymer Interaction and Miscibility and Its Impact on Drug Supersaturation in Aqueous Medium. Eur. J. Pharm. Biopharm. 2016, 107, 16–31. 10.1016/j.ejpb.2016.06.024. 27378287

[ref59] Lehmkemper K.; Kyeremateng S. O.; Heinzerling O.; Degenhardt M.; Sadowski G. Long-Term Physical Stability of PVP- and PVPVA-Amorphous Solid Dispersions. Mol. Pharmaceutics 2017, 14 (1), 157–171. 10.1021/acs.molpharmaceut.6b00763. 28043133

[ref60] Thakore S. D.; Akhtar J.; Jain R.; Paudel A.; Bansal A. K. Analytical and Computational Methods for the Determination of Drug-Polymer Solubility and Miscibility. Mol. Pharmaceutics 2021, 18 (8), 2835–2866. 10.1021/acs.molpharmaceut.1c00141. 34041914

[ref61] Lin X.; Hu Y.; Liu L.; Su L.; Li N.; Yu J.; Tang B.; Yang Z. Physical Stability of Amorphous Solid Dispersions: A Physicochemical Perspective with Thermodynamic, Kinetic and Environmental Aspects. Pharm. Res. 2018, 35 (6), 125 10.1007/s11095-018-2408-3. 29687226

[ref62] Rumondor A. C. F.; Stanford L. A.; Taylor L. S. Effects of Polymer Type and Storage Relative Humidity on the Kinetics of Felodipine Crystallization from Amorphous Solid Dispersions. Pharm. Res. 2009, 26 (12), 2599–2606. 10.1007/s11095-009-9974-3. 19806435

[ref63] Sun D. D.; Ju T. C. R.; Lee P. I. Enhanced Kinetic Solubility Profiles of Indomethacin Amorphous Solid Dispersions in Poly(2-Hydroxyethyl Methacrylate) Hydrogels. Eur. J. Pharm. Biopharm. 2012, 81 (1), 149–158. 10.1016/j.ejpb.2011.12.016. 22233548

[ref64] Borbás E.; Sinkó B.; Tsinman O.; Tsinman K.; Kiserdei É.; Démuth B.; Balogh A.; Bodák B.; Domokos A.; Dargó G.; Balogh G. T.; Nagy Z. K. Investigation and Mathematical Description of the Real Driving Force of Passive Transport of Drug Molecules from Supersaturated Solutions. Mol. Pharmaceutics 2016, 13 (11), 3816–3826. 10.1021/acs.molpharmaceut.6b00613. 27611057

