# Supplementary material for: Kinetic and Thermodynamic Interplay of Polymer-Mediated Liquid–Liquid Phase Separation for Poorly Water-Soluble Drugs
Source: Mol Pharm. 2024 May 20;21(6):2878–93. doi: 10.1021/acs.molpharmaceut.4c00033 (PMC11151203; doi:10.1021/acs.molpharmaceut.4c00033)
Supplement: Supplementary file 1 — mp4c00033_si_001.pdf [file mp4c00033_si_001.pdf]

The Kinetic and Thermodynamic Interplay of Polymer-mediated Liquid-Liquid Phase Separation for Poorly Water-soluble Drugs

Support information

Kaijie Qian[1], Lorenzo Stella[2], Fanjun Liu[1], David S. Jones[1], Gavin P. Andrews[1], Yiwei Tian[1*]

[1] School of Pharmacy, McClay Research Centre, Queen's University Belfast, 97 Lisburn Road, Northern Ireland BT9 7BL, UK.

[2] School of Mathematics and Physics, Queen's University Belfast, University Road, Belfast BT7 1NN, UK.

[3] School of Chemistry and Chemical Engineering, Queen's University Belfast, Stranmillis Road, Belfast BT9 5AG, UK.

Corresponding Author: Dr Yiwei Tian, y.tian@qub.ac.uk

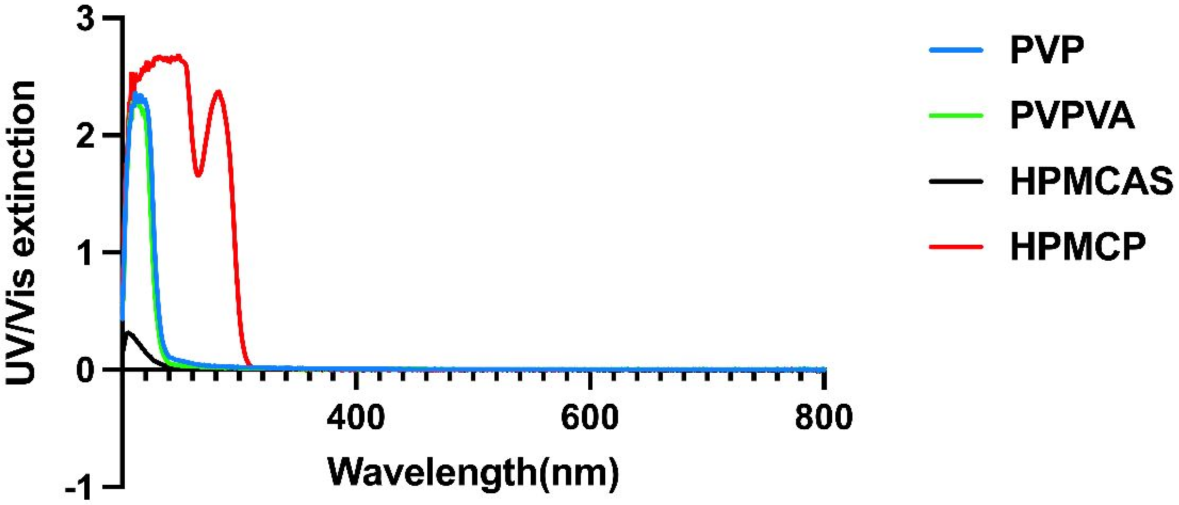

Figure S1. UV/Vis spectra at the wavelength range from 200 to 800 nm of polymers with 1mg/mL concentration. Blue, green, black, and red curves represented the spectra of polymers PVP, PVPVA, HPMCAS, and HPMCP.

Table S1. The parameters for construction of the drug-polymer-water phase diagrams

Polymers	Molecular volume/unit (cm3/mol)	Average Molecular volume/molecule (cm3/mol)	solubility parameter
HPMCP	188.28	37421.875	25.63
HPMCAS	494.16	14396.88716	19.76
PVP	92.58	8083.333333	23.26
PVPVA	179.3	46747.96748	26.31
Drug	Molecular volume/unit	solubility parameter	
Celecoxib	259.44	22.09	
Water	18	44.39	
	F-H interaction parameters mean	F-H interaction parameters lower values	
HPMCP-water	1.57	0.31	
HPMCAS-water	2.35	0.47	
PVP-water	0.49	0.11	
PVPVA-water	0.98	0.19	
celecoxib-water	3.6	2.21	
celecoxib-HPMCP	0.09	0.02	
celecoxib-HPMCAS	0.04	0.09	
celecoxib-PVP	0.011	0.029	
celecoxib-PVPVA	0.13	0.026	
For modelling of the ternary phase diagram	MW	Density	
HPMCP	47900	1.28	
HPMCAS	18500	1.285	
PVP	9700	1.2	
PVPVA	57500	1.23	

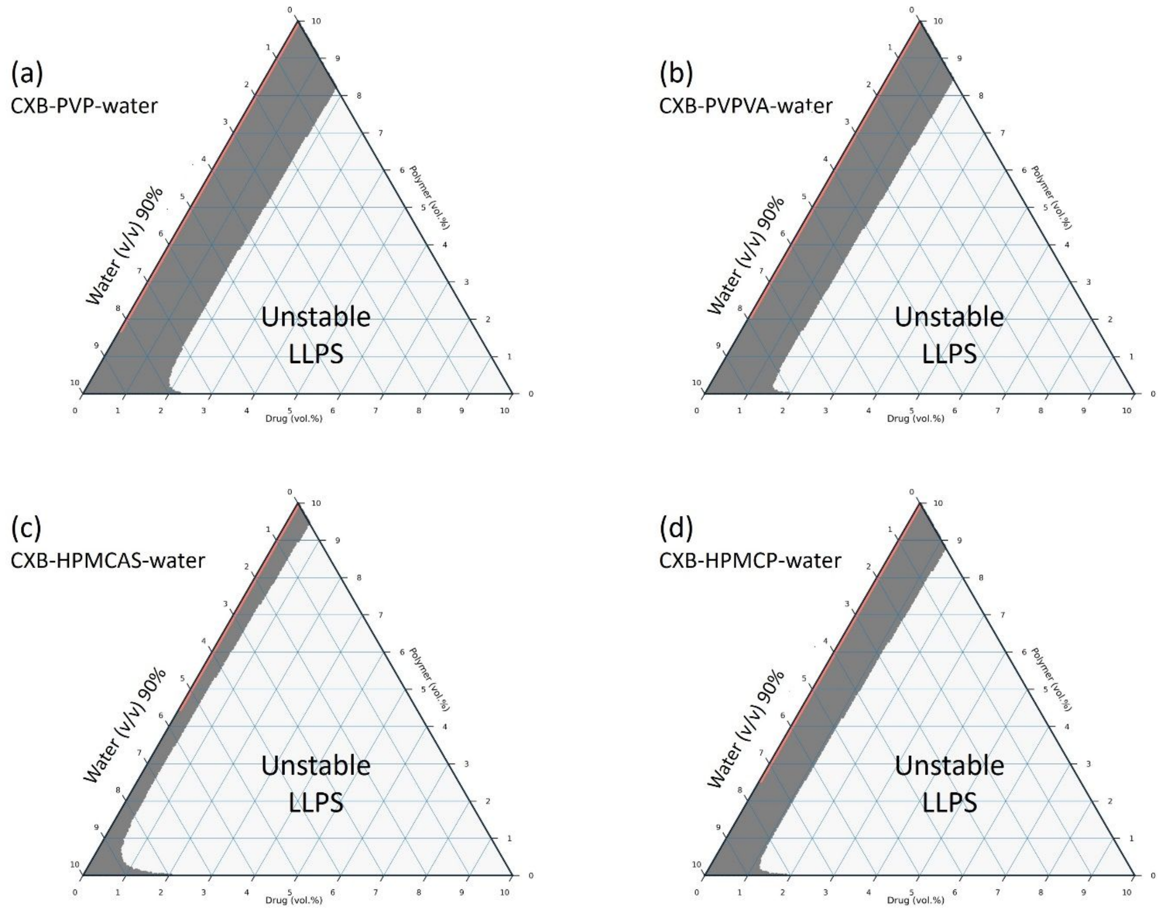

Figure S2. Construction of ternary phase diagrams for drug/polymer/water at high water compositions; the low values of F-H interaction parameters in Table S1 were used for the constructions.

Selection of the UV/Vis wavelength for detecting CXB LLPS onset point.

The spectrum region between 200 to 400 nm reveals the significant extinction of CXB due to UV absorption, with a clear absorption peak at a wavelength range of 230 to 300 nm and a maximum extinction value at 252 nm (Figure S2). The UV extinction values of the CXB-PVPVA sample at 252 nm were plotted as a function of CXB concentrations. In CXB-PBS solutions, the UV extinction profiles may be influenced by both absorption and scattering. In CXB-MeOH solutions, absorption becomes the sole factor. However, no significant difference could be observed at the wavelength of 252 nm between the CXB-PBS and CXB-MeOH solutions. The result indicated that at the wavelength of 252 nm, absorption is the main factor influencing the overall UV extinction of CXB solutions. In addition, the crystalline solubility of the fast crystallisation drug CXB is 1.50-1.58 μg/mL (Abu-Diak et al., 2011; Ilevbare et al., 2013). The crystallisation and/or phase separation would occur at high drug concentrations (up to 50 μg/mL in this experiment). The similar UV extinction-concentration profiles of CXB-PBS and CXB-MeOH solutions indicated that the absorption of electromagnetic radiation happens not only for the free drug molecules in solutions but also for the drug in nanocrystals. The UV extinctions at wavelengths higher than 300 nm differed from those at low wavelengths. No extinction was observed at 360 nm for the CXB-MeOH solutions (black square symbols), indicating the absorption of CXB was absent at this wavelength. In the CXB-PBS solution, the UV extinction was absent at low drug concentrations (0 to 20 μg/mL). When drug concentration increases to 30 μg/mL, additional extinction was observed at 360 nm

regions caused by the particle scattering (orange circle symbols, Figure S2d). Dash lines were regression curves derived from various drug concentration samples.

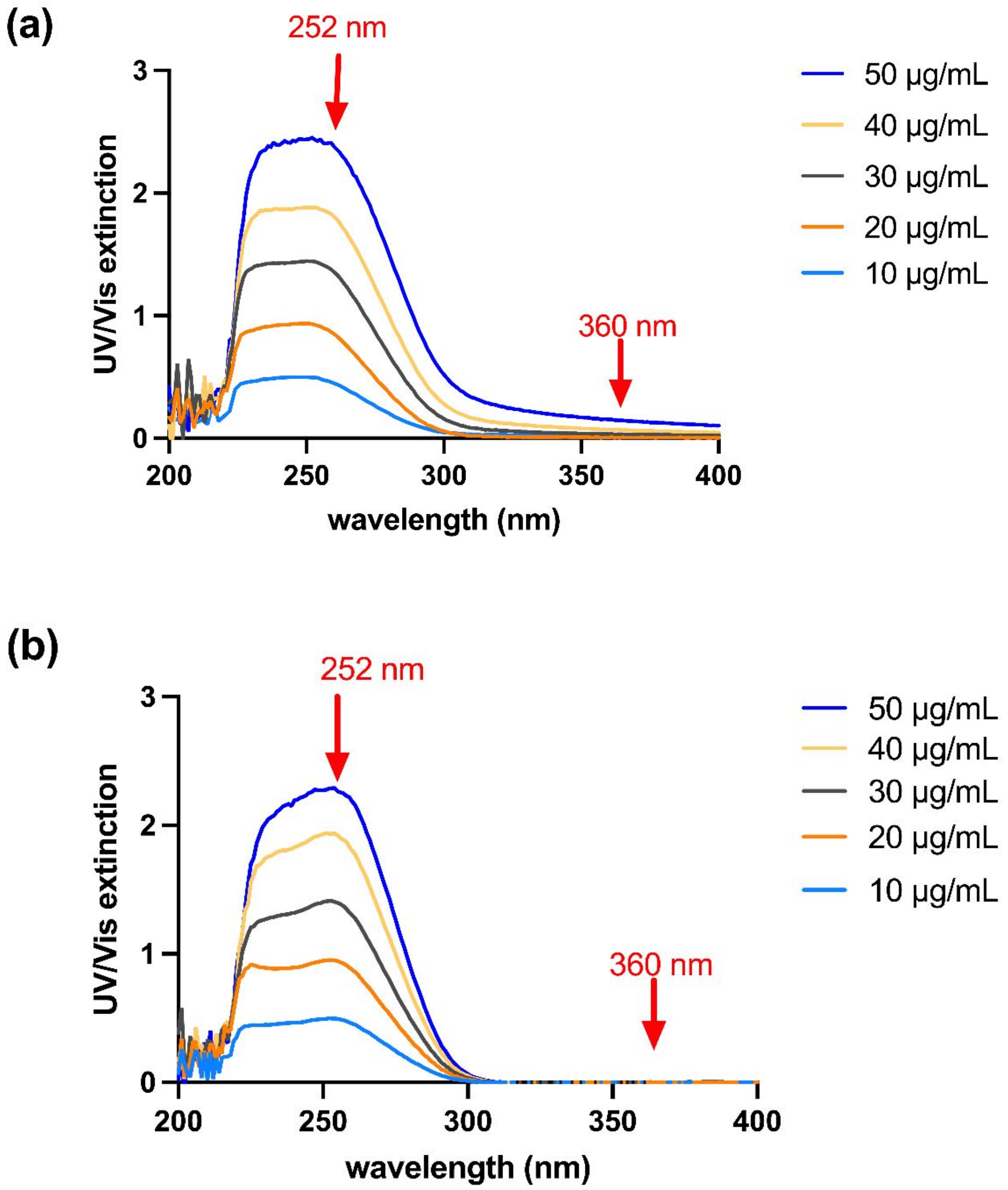

(c)

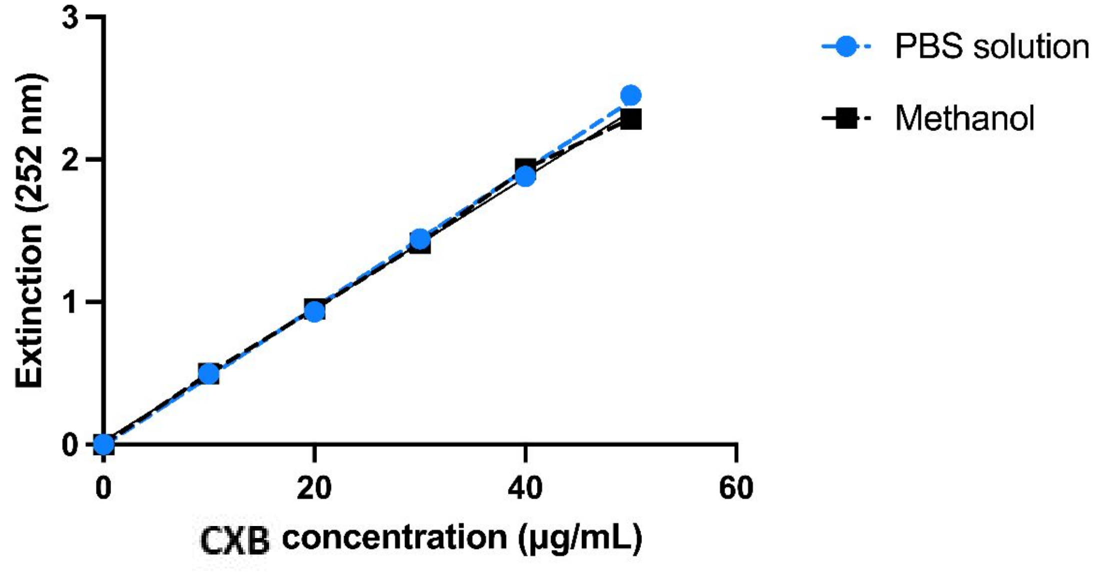

(d)

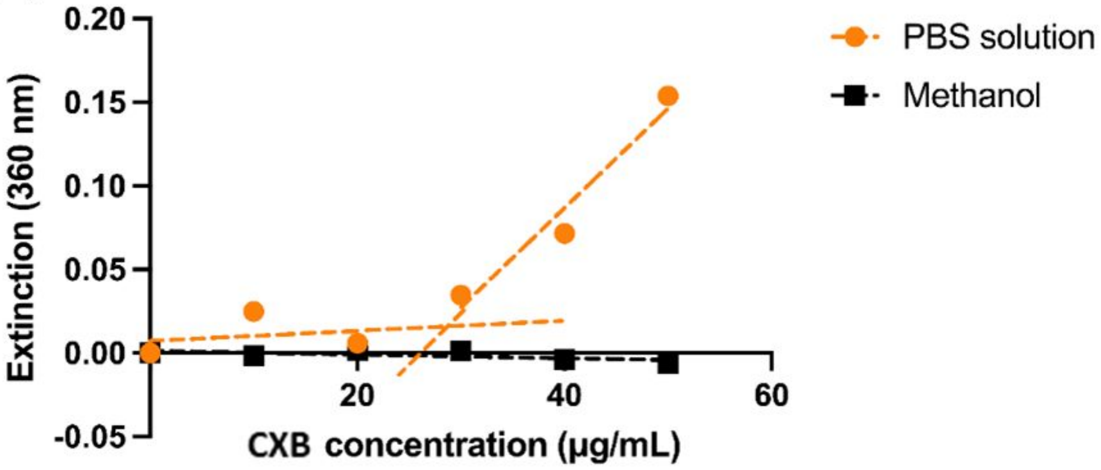

Figure S2. UV extinction spectra of (a) CXB-PBS solution (pH=7.4) and (b) CXB-MeOH with the drug concentration range of 10 to 50 μg/ml (from bottom to top, in 10 μg/ml interval), at the wavelength range of 200 to 400 nm. PVPVA was added into the PBS solution and MeOH to suppress the drug precipitation. CXB solution extinction at the wavelength of (c) 252 nm and (d) 360 nm as a function of the drug concentration (error bars were smaller than symbols). Circle and square symbols represented the extinction of CXB determined in the PBS and MeOH solutions, respectively. Dash lines represented regression curves.

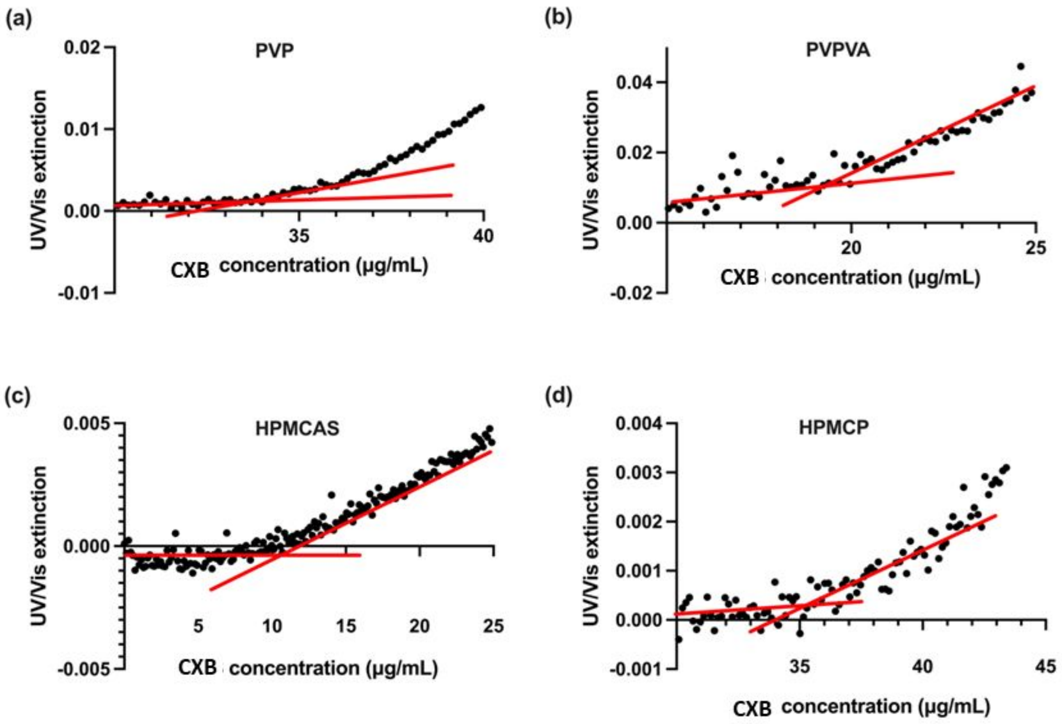

Figure S3. UV extinction profiles of CXB-polymer-water ternary systems as a function of CXB concentration (μg/mL), with the drug stock solution mixing rate of 1 mL/h. 1 mg/mL polymers of (a) PVP, (b) PVPVA, (c) HPMCAS, and (d) HPMCP were pre-dissolved in the pH7.4 PBS buffer at 37 ℃. Red lines represented least-squares regression curves.

Videos of the CXB-polymer-water LLPS can be found in the following links:

CXB – HPMCAS TIRFM: https://youtu.be/2C3YEFunhv4?si=aSbigGX58Cr2PkEn

CXB – HPMCAS polarized: https://youtu.be/xHuuLjqV0u4?si=5SP-e7Ds5Zd2ExcT

CXB – PVP TIRFM: https://youtu.be/mlWBwcKnMOs?si=kmsabXBqXvvzYxRc

CXB – PVP polarized: https://youtu.be/1IhxVAXBqD4?si=q5BNxc33sGhDX0B8
